# Developmental neuroanatomy of the rosy bitterling *Rhodeus ocellatus* (Teleostei: Cypriniformes)—A microCT study

**DOI:** 10.1002/cne.25324

**Published:** 2022-04-25

**Authors:** Wenjing Yi, Thomas Mueller, Martin Rücklin, Michael K. Richardson

**Affiliations:** ^1^ Institute of Biology, University of Leiden Sylvius Laboratory Leiden the Netherlands; ^2^ Vertebrate Evolution, Development and Ecology Naturalis Biodiversity Center Leiden the Netherlands; ^3^ Division of Biology Kansas State University Manhattan Kansas USA

**Keywords:** brain ventricle, evolutionary development, neuroembryology, proliferation zone, prosomeric model

## Abstract

Bitterlings are carp‐like teleost fish (Cypriniformes: Acheilanathidae) known for their specialized brood parasitic lifestyle. Bitterling embryos, in fact, develop inside the gill chamber of their freshwater mussel hosts. However, little is known about how their parasitic lifestyle affects brain development in comparison to nonparasitic species. Here, we document the development of the brain of the rosy bitterling, *Rhodeus ocellatus*, at four embryonic stages of 165, 185, 210, 235 hours postfertilization (hpf) using micro‐computed tomography (microCT). Focusing on developmental regionalization and brain ventricular organization, we relate the development of the brain divisions to those described for zebrafish using the prosomeric model as a reference paradigm. Segmentation and three‐dimensional visualization of the ventricular system allowed us to identify changes in the longitudinal brain axis as a result of cephalic flexure during development. The results show that during early embryonic and larval development, histological differentiation, tissue boundaries, periventricular proliferation zones, and ventricular spaces are all detectable by microCT. The results of this study visualized with differential CT profiles are broadly consistent with comparable histological studies, and with the genoarchitecture of teleosts like the zebrafish. Compared to the zebrafish, our study identifies distinct developmental heterochronies in the rosy bitterling, such as a precocious development of the inferior lobe.

## Graphical abstract

Rosy bitterling brain at 165 hours postfertilization. Tissue boundaries, periventricular proliferation zones, and ventricular spaces are all detectable by microCT in this very early stage. Three‐dimensional visualization with microCT scanning protocols is extremely useful for the study of the rosy bitterling and provides an updating of imaging modalities for morphological and anatomical analyses in this nonmodel organism.

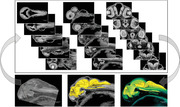

**Abbreviations**
III: oculomotor nerve
IV: trochlear nerve
V: trigeminal nerve
Va: valvula cerebeli
VI: abducens nerve
VII: facial nerve
IX: glossopharyngeal nerve
X: vagus nerve
ac: anterior commissure
AIS: anterior intraencephalic sulcus
Ccer: commissure cerebelli
CeP: cerebellar plate
Ch: notochord
DIV: trochlear decussation
DVe: diencephalic ventricle
E: epiphysis
EmT: eminentia thalami
fp: floor plate
fr: fasciculus retroflexus
GM: gray matter
Ha: habenula
Hc: caudal hypothalamus
Hi: intermediate hypothalamus
Hr: rostral hypothalamus
Hy: hypophysis
IL: inferior lobe of hypothalamus
le: lens
lfb: lateral forebrain bundle
lr: lateral rectus muscle
LR: lateral recess of hypothalamic ventricle
mlf: medial longitudinal fascicle
mou: mouth
M: Mauthner neurons
M1: pretectal migrated area
M2: posterior tubercular migrated area
M3: EmT migrated area
M4: telencephalic migrated area
MHB: midbrain‐hindbrain boundary
MV: median ventricle of the mesencephalic ventricle
MVS: median ventricular sulcus of the mesencephalic ventricle
N: region of the nucleus of the medial longitudinal fascicle
NIII: oculomotor nucleus
NIV: trochlear nucleus
NVa: rostral trigeminal motor neuron
NVII: facial motor neuron
NIn: interpeduncular nucleus
NVp: caudal trigeminal motor neuron
OA: occipital arch
OB: olfactory bulb
oc: optic chiasma
OC: otic capsule
OE: olfactory epithelium
of: oval fossae
OG: octaval ganglion (VIII)
on: optic nerve
ON: olfactory nerve
ot: otolith
P: pallium
pc: posterior commissure
pf: pectoral fin
Po: preoptic region
poc: postoptic commissure
PoR: preoptic recess
Pr: pretectum
PR: posterior recess of hypothalamic ventricle
ptc: posterior tubercular commissure
PTd: dorsal part of posterior tuberculum
PTh: prethalamus
PTM: posterior tectal membrane
PTv: ventral part of posterior tuberculum
r: rhombomere
re: retina
RL: rhombic lip
RVe: rhombencephalic ventricle
S: subpallium
SC: spinal cord
Sd: dorsal subpallium
sr: superior rectus muscle
Sv: ventral subpallium
T: midbrain tegmentum
tc: tela choroidea
Tel: telencephalon
TeO: tectum opticum
TeVe: tectal ventricle
Th: thalamus
TL: torus longitudinalis
tpc: tract of posterior commissure
TS: torus semicircularis
TVe: telencephalic ventricle
WM: white matter
Zli: zona limitans intrathalamica

## 1 INTRODUCTION

Bitterlings, a group of freshwater teleosts, have been established as valuable model species in behavioral, population, and evolutionary ecology due to their brood parasitic life history. Their peculiar lifestyle involves the laying of eggs by the bitterling in a host mussel, a phenomenon that has been recognized for more than a century (Boeseman et al., 1938; Chang, 1948; Duyvené de Wit, 1955; Kitamura et al., 2012; Mills & Reynolds, 2003; Olt, 1893; Reichard et al., 2007; Rouchet et al., 2017; Smith, 2016; Wiepkema, 1962). Noll (1877) was the first to show that the embryos of European bitterling (*Rhodeus amarus*) develop in the gill chamber of their host mussel. This location provides a sheltered environment which protects the developing embryos from potential predators (Aldridge, 1999; Liu et al., 2006; Reichard et al., 2007; Smith et al., 2004). We (Yi et al., 2021) have recently compared developmental sequences of the rosy bitterling (*Rhodeus ocellatus*) to the zebrafish (*Danio rerio*); the latter is a nonparasitic teleost which lays its eggs into the open water (Kimmel et al., 1995; Lawrence, 2007). That study confirmed the relative pre‐displacement of hatching and the relative delay of development of the pectoral fins in the bitterling.

The specialized ontogeny of the bitterling and its brood parasitic lifestyle make it a potentially interesting model for the study of developmental mechanisms underlying brain evolution. In this study, we generated an atlas of the developing bitterling brain as a reference for cross‐species comparisons. To build a foundation for such comparisons, we related the bitterling brain development with published data of the zebrafish. The comparison to the zebrafish is useful for two reasons: first, the zebrafish is firmly established as a genetic model system that has been most thoroughly investigated with regard to embryonic, postembryonic, and larval stages (Mueller & Wullimann, 2003, 2016; Mueller et al., 2006; Wullimann, 2009; Wullimann & Knipp, 2000; Wullimann & Mueller, 2004); second, both the bitterling and the zebrafish belong to the group of carp‐like (cyprinid) teleosts with very similar adult brain anatomy yet, as we show, distinct developmental heterochronies. A developmental stage atlas of the bitterling brain as visualized in this study, provides a foundation to examine the molecular mechanisms underlying these heterochronies.

In the vertebrates, the central nervous system, including its anteriormost part, the brain, develops from the neural tube (Richardson & Wright, 2003; Schmitz et al., 1993; Wullimann, 2009). In teleosts, including bitterlings, the development of the neural tube involves secondary neurulation (Schmitz et al., 1993). The definitive neural tube is filled with cerebrospinal fluid (CSF) (Lowery & Sive, 2009). The neural tube caudal to the brain is the spinal cord, and has a narrow lumen called the central canal. The brain has an inflated, irregular lumen which forms a series of brain ventricles (Korzh, 2018). In general, the vertebrate brain consists of four parts: (1) the secondary prosencephalon, (2) the diencephalon, (3) the mesencephalon, and (4) the rhombencephalon. Correspondingly, the brain ventricular system has been divided into the fourth ventricle (the lumen of the rhombencephalon); the mesencephalic ventricle (MV); the third ventricle (the lumen of diencephalon proper); and the prosencephalic ventricle (including the telencephalic ventricle [TVe] and the hypothalamic part of the classic diencephalic ventricle (DVe); Nieuwenhuys & Puelles, 2016).

In this study we used the prosomeric model of Puelles and Rubenstein (2003) for dividing neuromeres in the secondary prosencephalon as well as for defining other brain divisions. In general, the prosomeric model alongside its recognition of longitudinal zones and transverse neuromeres forms a powerful paradigm for vertebrate cross‐species comparisons. One reason for this is that the model is based on conserved molecular and developmental characteristics that allow a consistent demarcation of the central nervous system (CNS) into developmental units (morphogenetic entities) along the neuraxis of a range of vertebrate species. Applying the prosomeric model for analyzing the development of the zebrafish brain, it has been demonstrated that cellular processes, i.e., proliferation, migration, and differentiation, can be used to define prosomeric units in teleosts (Mueller & Wullimann, 2003; Mueller & Wullimann, 2016; Mueller et al., 2006; Wullimann, 2009; Wullimann & Knipp, 2000; Wullimann & Mueller, 2004).

The prosomeric model analyzes brain regions along the general brain axis and according to the mediolateral extent of brain regions. Longitudinally, the neural tube has four compartments divided by the sulcus limitans that were first defined by Wilhelm His, Sr. (His Wilhelm, 1895); reviewed by Puelles (2019). The four compartments are—the roof plate dorsally, the alar plate dorsolaterally, the basal plate ventrolaterally, and the floor plate ventrally.

According to Nieuwenhuys and Puelles (2016), the rhombencephalon consists of 12 neuromeres (rhombomeres, isthmus or r0 plus r1‐r11) in longitudinal series. In cyprinids like the Goldfish (*Carassius auratus*), the rostral rhombomeres two to six (r2‐r6) correspond to the mammalian pons, whereas the caudal rhombomeres seven to eight (r7‐8) correspond to the mammalian medulla oblongata (Gilland et al., 2014; Rahmat & Gilland, 2019). The rostral rhombomeres r2‐r6 form clearly segmented neural clusters while the caudal r7‐r8 lack a precise morphological delineation (Ma et al., 2009). The zebrafish r8 is twice as large as the rostral rhombomeres, composed by multiple crypto‐ or pseudo‐rhombomeres that can only be delimited molecularly as similar to the avian brain (Cambronero & Puelles, 2000). The rostral subdivision of neuromeres is more complex, because bending of the neural tube (the cephalic flexure, Hauptmann & Gerster, 2000; Mueller & Wullimann, 2009) has made the definition of the longitudinal neuraxis more difficult (Mueller & Wullimann, 2009; Puelles, 2019; Vernier, 2017; Wullimann & Rink, 2002).

Here, we visualize the development of the rosy bitterling (*Rhodeus ocellatus*) brain using micro‐computed tomography (microCT, x‐ray microscopy or μCT) with a specific focus on developmental regionalization and brain ventricular organization. MicroCT is a widely used high‐resolution, non‐destructive, three‐dimensional (3D) imaging technique (Babaei et al., 2016; Metscher, 2009a) that just recently has been introduced to study development (Wong et al., 2015). The contrast of CT scans is based on absorption of x‐ray radiation passed through the sample. Conventionally, highly mineralized structures such as bones and teeth have higher attenuation coefficient (CT value or Hounsfield units) and are brighter and easier to recognize than soft tissues. For the visualization of the latter, a treatment with contrast agents is needed. In this work, we used phosphotungstic acid (PTA; Metscher, 2009b), which allows discrimination of soft tissues such as muscles, nerves, and blood vessels. In this study, we extend MicroCT techniques to the developing fish brain. Our goal is to establish microCT and 3D visualizations as complementary methods for cross‐species comparisons of structural characteristics of both developing and mature brains. In fact, our results indicate that microCT is useful to quantitatively analyze, for example, ventricular spaces and white matter versus proliferative and postmitotic cell masses.

## 2 MATERIALS AND METHODS

### 2.1 Sample preparation

Embryos of six developmental stages of 135, 150, 165, 185, 210, 235 hours postfertilization (hpf; Table 1) were obtained by in vitro fertilization following the method of Nagata and Miyabe (1978). Embryos were staged according to Yi et al. (2021). Embryos were incubated in a temperature‐controlled incubator (22.5 ± 1°C) and fixed in 3% paraformaldehyde (pFA) and 1% glutaraldehyde (GA). Digital microphotographs of fixed samples were obtained with a charge‐coupled device (CCD) camera connected to a stereo microscope (Nikon SMZ1500). For x‐ray contrast enhancement, embryos were stained for at least 24 h in 0.3% PTA dissolved in 70% ethanol. Samples were then brought back to 70% ethanol without PTA and mounted in low‐melting point agarose for non‐shift scanning in pipette tips.

**TABLE 1:** Sample information and microCT scanning parameters

Age (hpf)	Stage name	Pixel size (μm)	Voltage (keV/W)	Exp. time (sec.)	Intensity
135	2‐ovl	2.19	40/3	4	5,000‐9,000
150	1‐ovl	2.0615	40/3	4.5	5,000‐10,000
165	1‐ovl/pec‐bud	0.9765	40/3	17	5,000‐8,500
185	pec‐bud	0.9989	80/7	3	5,000‐6,300
210	high‐pec	1.4582	40/3	9.5	5,000‐10,000
235	long‐pec	1.4299	40/3	8.5	5,000‐10,000

*Note*: For each stage, we scanned at least two specimens. The stage names follow Yi et al. (2021). For the pec‐bud stage at 185 hpf, we also tried a lower resolution scan with the voltage set to 40/3 keV/W. We found no practical difference in image quality between scans at 80/7 keV/W and 40/3 keV/W. Therefore, we decided to use the less time‐consuming option for this stage, namely scanning with the higher voltage but shorter exposure time.

Abbreviations: Dev, developmental; Exp, exposure; hpf, hours postfertilization; keV, kiloelectron volt.

### 2.2 MicroCT scanning

Attenuation‐based microtomographic images were acquired using a Zeiss Xradia 520 Versa 3D X‐ray microscope, with the x‐ray tube voltages source set at 80/7 or 40/3 keV/W (keV: kiloelectron volts). A thin LE1 filter was used to avoid beam‐hardening artifacts. During the CT scanning, the sample was placed on a rotation table and projection images were acquired over an angular range of 180 degrees. To obtain high resolution images, a CCD optical objective with 4× was applied in the scan. Images were acquired with voxel (volumetric 3D pixels after reconstruction) sizes of 1‐1.5 μm, and tomographic reconstructions were made with the resident software XMReconstructer (Carl Zeiss X‐ray Microscopy Inc., Pleasanton, CA). Reconstructed images were exported as TIFF and loaded into Avizo version 9.5 (Thermo Fisher Scientific, https://www.fei.com/software/amira-avizo/) for 3D visualization.

### 2.3 3D visualization

The reconstructed volume was viewed “slice by slice” as virtual sections using the Slice module in the Avizo software (Version: 9.5; Thermo Fisher Scientific). A computational module “Resample transformed image” was applied to register images to the orthogonal direction of the anatomical axis. Ortho View was used for interactive orthogonal views in *xy, yz*, and *xz* axis simultaneously. The Volume Rendering module was used for 3D view. Values of colormap and opacity degree were optimized in the settings of rendering. Scalebars were added using the Scalebars module.

### 2.4 Annotations on 2D slices

**FIGURE 1:**
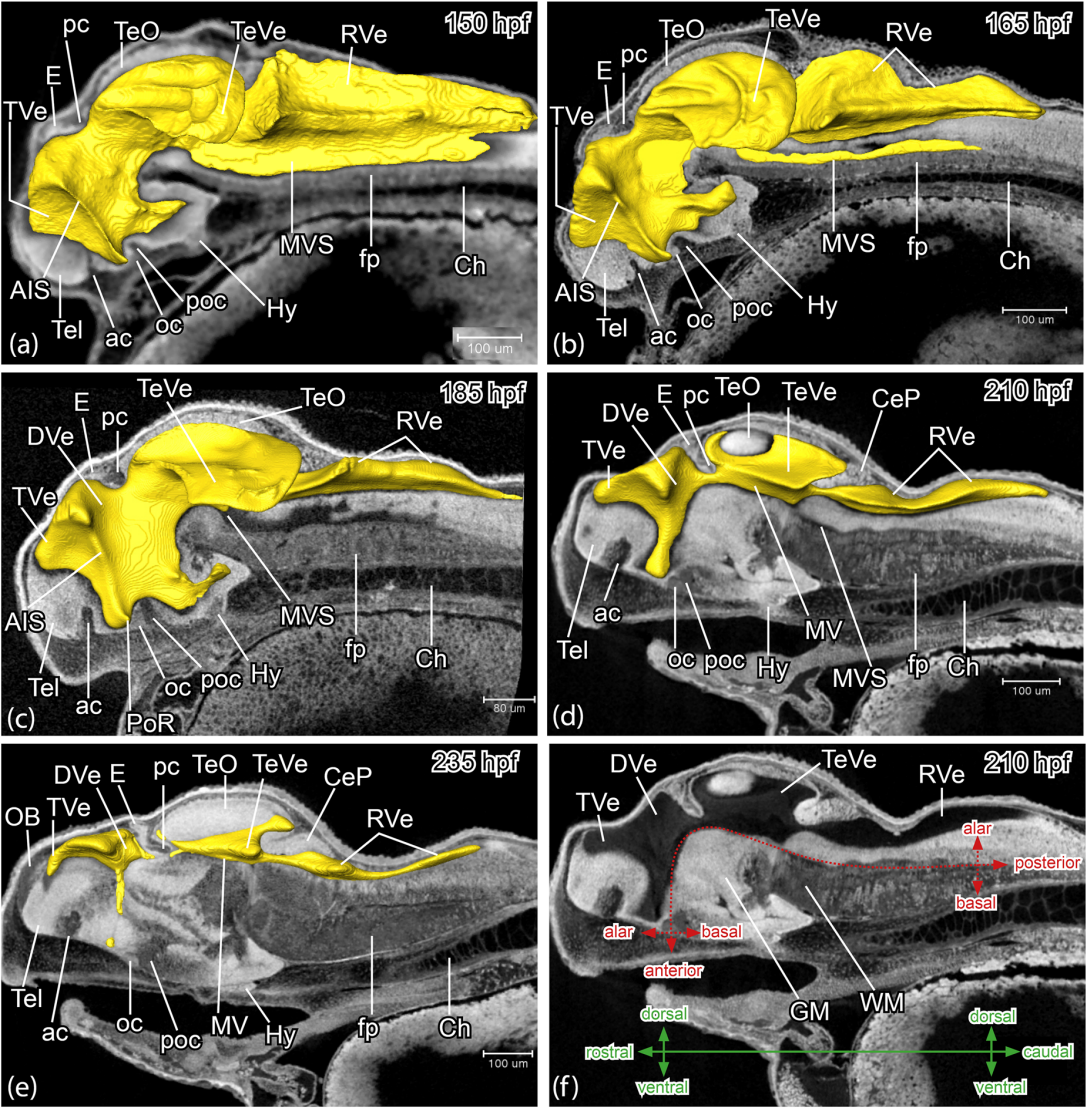
*Rhodeus ocellatus*, development of the brain ventricular system, micro‐computed tomography (microCT) images, dorsal to the top, head to the left. (a‐e) Virtual midsagittal sections overlayed with surface view of manually segmented brain ventricles, and showing the distinct ridge of the anterior intraencephalic sulcus (AIS) and progressive compartmentalization of the ventricular system (a‐e). (f) Virtual midsagittal section illustrating the black lumen of the brain ventricle, a bright white periventricular layer of gray matter (GM), and a gray peripheral layer of white matter (WM). The red dotted line indicates the neuraxis, with anterior, posterior, alar, and basal topological direction for the curved axis. The green axes refer to the linear axial system apply to the brain parts with the terms rostral, caudal, dorsal, and ventral. The (a) stage 1‐ovl, 150 hours postfertilization (hpf). (b) STAGE 1‐ovl/pec‐bud, 165 hpf. (c) stage pec‐bud, 185 hpf. (d, f) stage high‐pec, 210 hpf. (e) Stage long‐pec, 235 hpf. For annotations, see list of abbreviations. Scale bars = 100 μm (a, b, d, e. f); 80 μm (c)

To generate a developmental atlas, serial virtual transverse sections were taken in rostrocaudal sequence from the olfactory bulb to the medulla oblongata. The section plane was set parallel to the deep ventricular sulcus between telencephalon and diencephalon (the anterior intraencephalic sulcus or AIS). To keep a consistent prosomeric axis annotation, we visualized the transverse section from the rostral to caudal, the coronal section from dorsal to ventral, the sagittal section from medial to lateral along the general body axis (Figure 1f). Labels were added to each section in the Adobe InDesign software (Version: 15.0.2; Adobe Systems Inc., San José, California). For anatomical terms, see the list of abbreviations. In general, and to facilitate cross‐species comparisons, we adopted anatomical terms from the *Atlas of Early Zebrafish Brain Development* (Mueller & Wullimann, 2005, 2016).

### 2.5 Segmentation of brain ventricles

Segmentation of the brain ventricle was conducted in Avizo in two steps. First, a rough segmentation based on grayscale threshold was achieved semi‐automatically and polished by using Smooth labels and Remove islands filters. These segmentation results were checked slice‐by‐slice and were corrected manually. The Generate Surface module was used to extract surfaces from the segmentation results. Brain ventricles were colored in yellow using the Surface view module, while the rest of the cranial tissues were in semi‐transparent using the Volume rendering module. The segmented model of the brain ventricles was captured in dorsal, ventral, lateral, and rostral views and saved in TIFF format, annotated in Adobe InDesign.

## 3 RESULTS

We studied the developmental stages of 165, 185, 210, 235 hpf of the brain in the rosy bitterling (*Rhodeus ocellatus*) using microCT. Throughout this study we have used the stage table of development of the rosy bitterling generated by Yi et al. (2021). To avoid confusion regarding the anatomical orientation, we follow Herget et al. (2014), and use the term rostral, caudal, dorsal, and ventral as in classical descriptions for the linear axial system of the embryonic body. For outlining topological relationships, we have used the terms anterior, posterior, alar, and basal as alternatives (Figure 1f). With respect to the neuromeres, anterior and posterior are defined relative to the neural axis, which, because its flexure, is not necessarily the same as the primary body axis. We divided the results into two sections: first, we illustrated the development of the ventricular system; second, we compiled a developmental atlas of the bitterling brain.

### 3.1 Development of the brain ventricular system of the rosy bitterling

#### 3.1.1 General description

The grayscale values we observed on virtual microCT sections depend on certain properties of the tissue, such as dye precipitation, tissue density, and cell type. In the brain, the gray matter generally appeared brighter than the white matter (e.g., compare the telencephalon (Tel) and anterior commissure (ac) in Figure 1). The white matter, with its myelinated axons and tracts, yielded grayscale values typical of low‐density soft tissues (Figure 1, ac, postoptic commissure [poc], posterior commissure [pc]). In contrast, the brain ventricle, essentially a hollow space filled with CSF, showed a lower density than the gray or white of the brain. It was visible in CT scans as the darkest part (Figure 1a‐f, rendered in yellow color in Figure 1a‐e). Therefore, the radiological appearance of the brain presented itself as a three‐partitioned structure each separated by clear boundaries: (1) a black lumen (ventricle), (2) a bright white periventricular layer of gray matter, and (3) a gray peripheral layer of white matter (Figure 1f).

**FIGURE 2:**
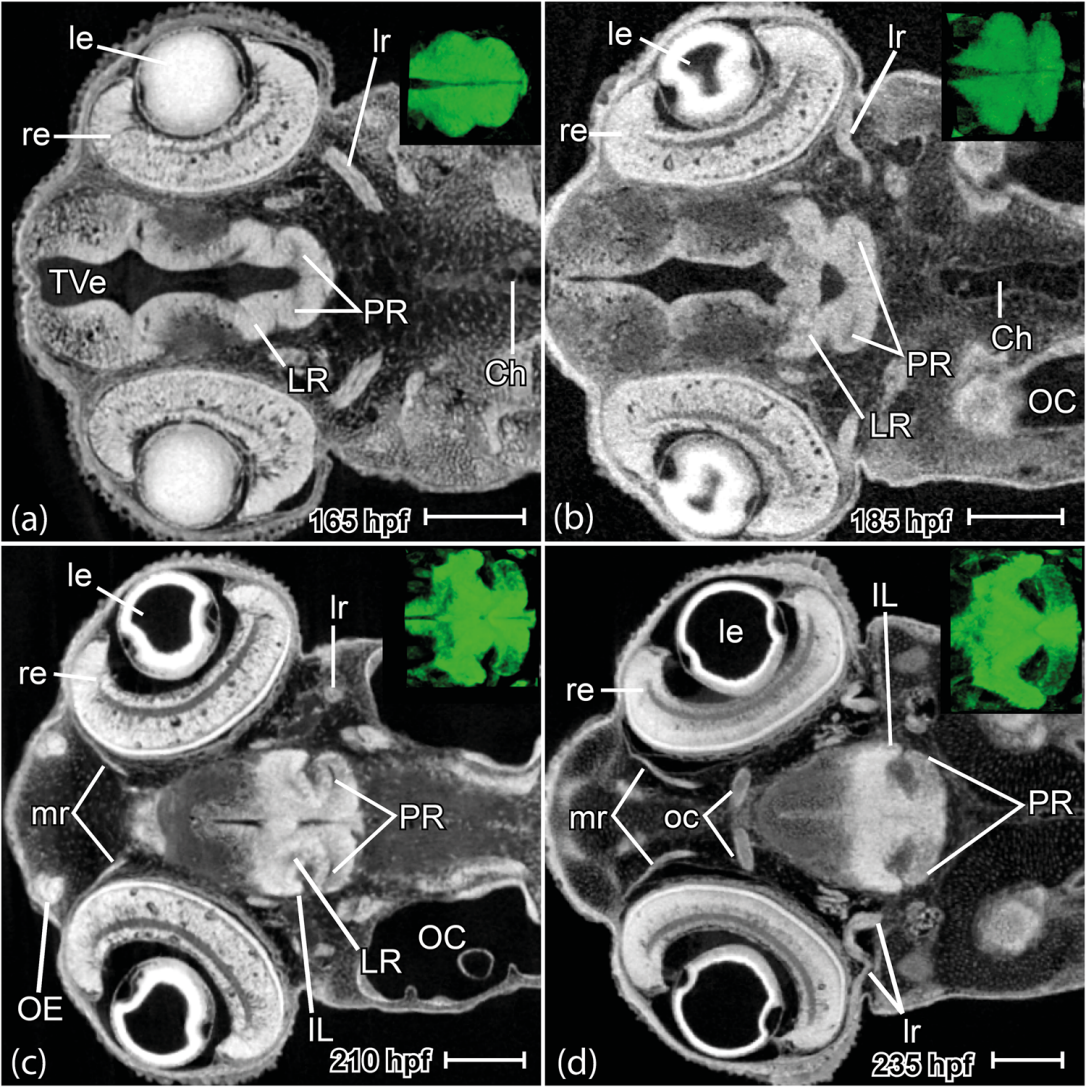
*Rhodeus ocellatus*, development of the lateral recess and posterior recess in the hypothalamus region. Virtual coronal sections, head to the left. At the right upper corner is the three‐dimensional (3D) volume rendering of the hypothalamus region seen from the dorsal side. (a) Stage 1‐ovl/pec‐bud, 165 hours postfertilization (hpf). (b) Stage pec‐bud, 185 hpf. (c) Stage high‐pec, 210 hpf. (d) Stage long‐pec, 235 hpf. For annotations, see list of abbreviations. Scale bars = 100 μm

The forebrain ventricle is divided dorsally by the position of the AIS into the anterior TVe and the posterior DVe proper (or the third ventricle; Figure 1). During the eversion of the TVe, the dorsal part of the AIS showed concomitant enlargement (Figure 1). Around the preoptic recess (PoR), the optic recess region (ORR) was recognizable, bordered by the ac and poc (see locations of ac, poc, and PoR in Figure 1c). The hypothalamic ventricle is topographically caudal (topologically basal) to the ORR, and topologically anterior to the third ventricle, including the lateral recess (LR) and the posterior recess (PR; Figure 2).

**FIGURE 3:**
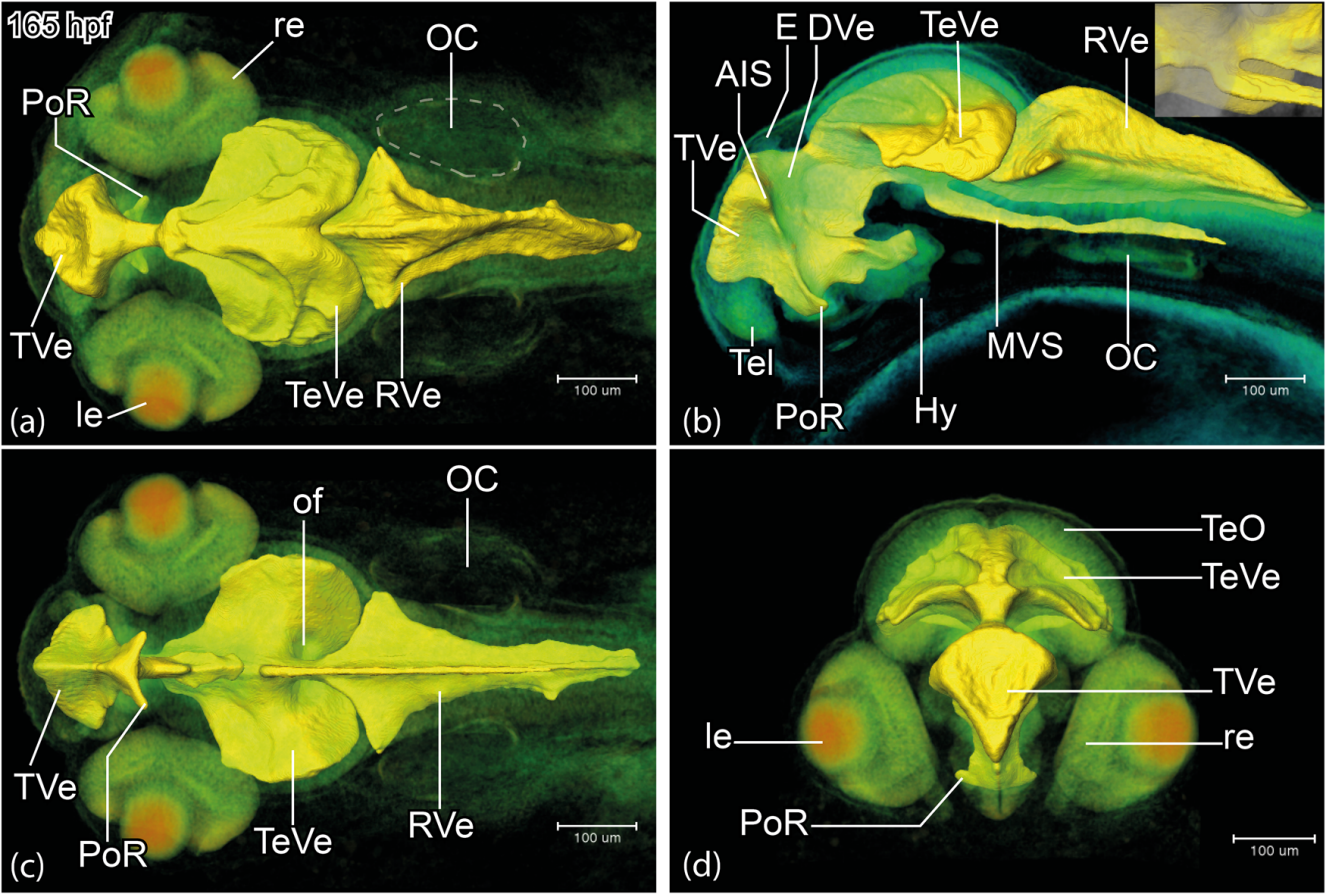
*Rhodeus ocellatus*, brain ventricular system at the stage 1‐ovl/pec‐bud, 165 hours postfertilization (hpf). (a‐d) microCT images, the pseudocolour volume rendering of the head region overlayed with a surface view of the manually‐segmented brain ventricles. (a‐d) Dorsal, lateral, ventral, and rostral views, respectively. The inset in the upper right corner in (b) illustrates the median ventricular sulcus (MVS) branches off from the ventral bottom of the median ventricle of the mesencephalon. For annotations, see list of abbreviations. Scale bars = 100 μm

The lumen of the mesencephalon ventricle contains the paired tectal ventricle (TeVe), the median ventricle (MV), and the median ventricular sulcus (MVS) (Puelles, 2019). The TeVe projects dorsolaterally from the MV (Figure 3a), covered by the subarea of the alar plate of the midbrain (optic tectum). A transiently visible MVS branches off from the ventral bottom of the MV and shows separately at the floor plate area (Figure 3b, inset in the upper right corner). The MV extends caudally to the rhombencephalic ventricle (RVe or the fourth ventricle; Figure 3; García‐Lecea et al., 2017; Korzh, 2018).

#### 3.1.2 STAGE 1‐ovl/pec‐bud, 165 hpf

In the rosy bitterling, the ventricular inflation is completed at stage 165 hpf (compare Figure 1a and b). The RVe is diamond‐shaped (rhombic) in dorsal and rostral aspects (Figure 3a and c). The bilateral TeVes resemble scallop shells (Figure 3a). The TVe is triangular in rostral aspect (Figure 3d). A pair of oval fossae (of) appear caudal‐ventral to the midbrain (Figure 3c). These fossae are occupied by the rostral cerebellar thickenings, which develop into the valvula cerebelli of the adult (Wullimann & Knipp, 2000). The LR and the PR of the hypothalamic ventricle are in shallow groves (Figure 2a).

#### 3.1.3 STAGE pec‐bud, 185 hpf

**FIGURE 4:**
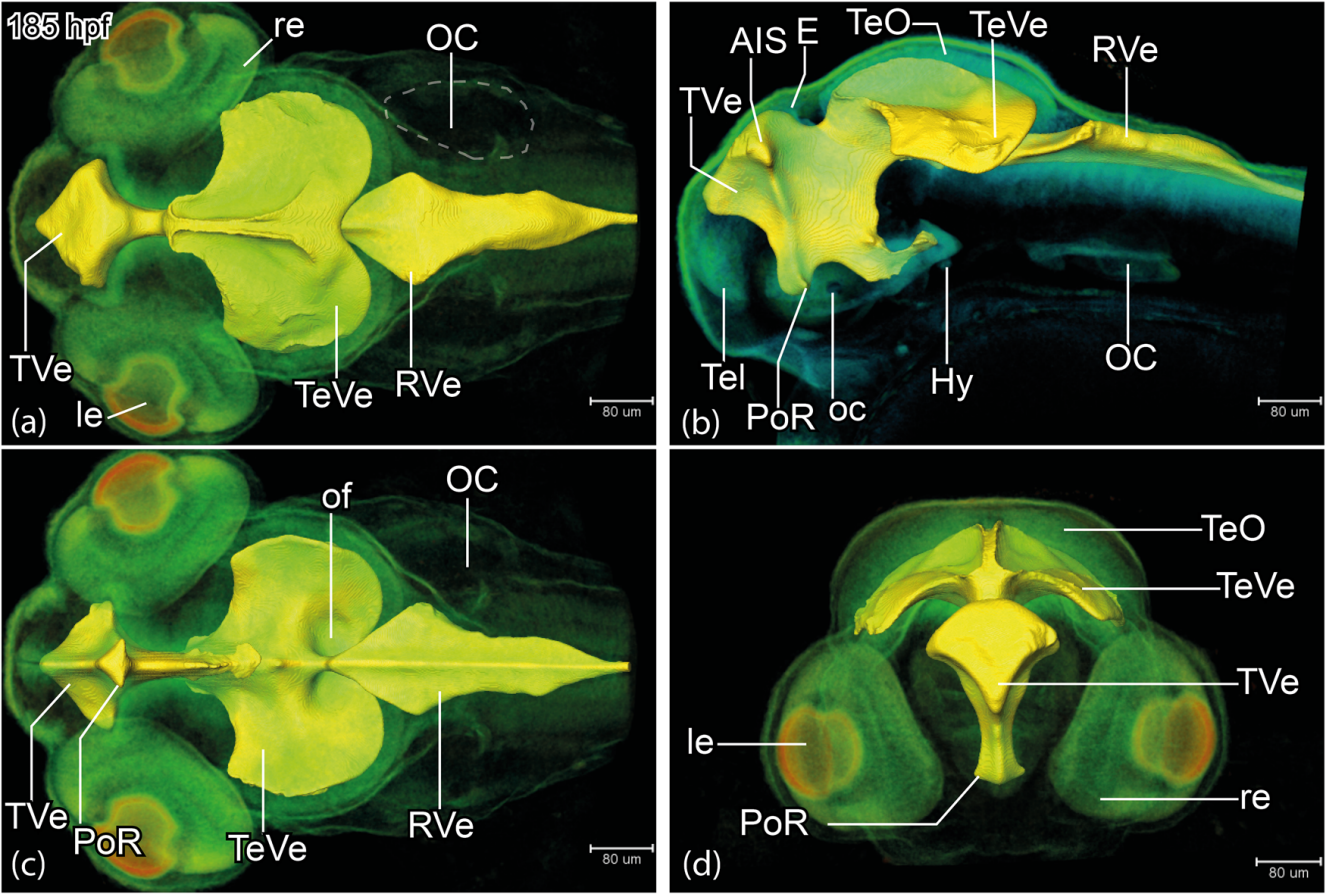
*Rhodeus ocellatus*, brain ventricular system at the stage pec‐bud, 185 hours postfertilization (hpf). (a‐d) microCT images, the pseudo‐colour volume rendering of the head region overlayed with a surface view of the manually segmented brain ventricles. (a‐d) Dorsal, lateral, ventral, and rostral views, respectively. For annotations, see list of abbreviations. Scale bars = 80 μm

Width of the RVe decreases to less than half of the TeVe width (Figure 4a). The RVe gradually flattens along its dorsal‐ventral axis (compare Figures 3b and 4b). The dorsal surface of the TeVe appeared smoother due to a further developed optic tectum (mammalian superior colliculus) and torus semicircularis (mammalian inferior colliculus; Figure 4a). There is a deep midline ridge that separates left and right TeVe (Figure 4a and d). The PoR is compressed and reduced in width (compare Figures 3c and 4c). The oval fossae (of) enlarges with the growth of the rostral cerebellar thickening (compare Figures 3c and 4c). The LR and PR extend outward and become observable (Figure 2b).

#### 3.1.4 STAGE high‐pec, 210 hpf

**FIGURE 5:**
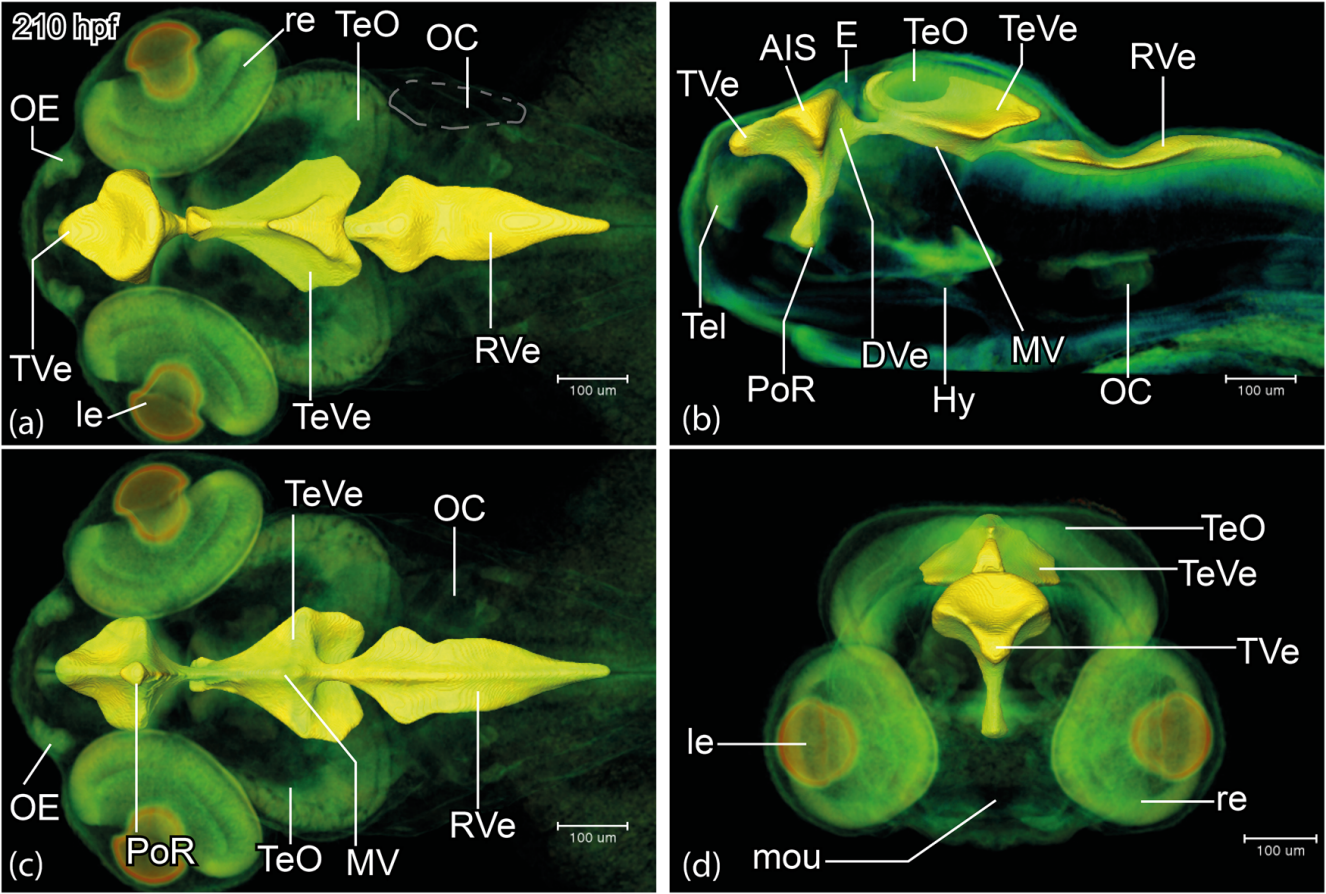
*Rhodeus ocellatus*, brain ventricular system at the stage high‐pec, 210 hours postfertilization (hpf). (a‐d) microCT images, the pseudocolour volume rendering of the head region is overlayed with a surface view of the manually segmented brain ventricles. (a‐d) Dorsal, lateral, ventral, and rostral views, respectively. For annotations, see list of abbreviations. Scale bars = 100 μm

Compared to earlier stages, the brain ventricles appeared compressed at 210 hpf (compare Figures 4a and 5a). However, the dorsal ventricle of the AIS appeared expanded, probably due to the eversion of the TVe. The tectal lobes grow larger and adhesion between the right and left lobes is emergent in the midline; therefore, the deep midline ridge of the TeVe appeared compressed rostrally (Figure 5b). The LR extends basalward to flange the PR; around the LR forms the inferior lobe (IL, Figure 2c; Bloch et al., 2019).

#### 3.1.5 STAGE long‐pec, 235 hpf

**FIGURE 6:**
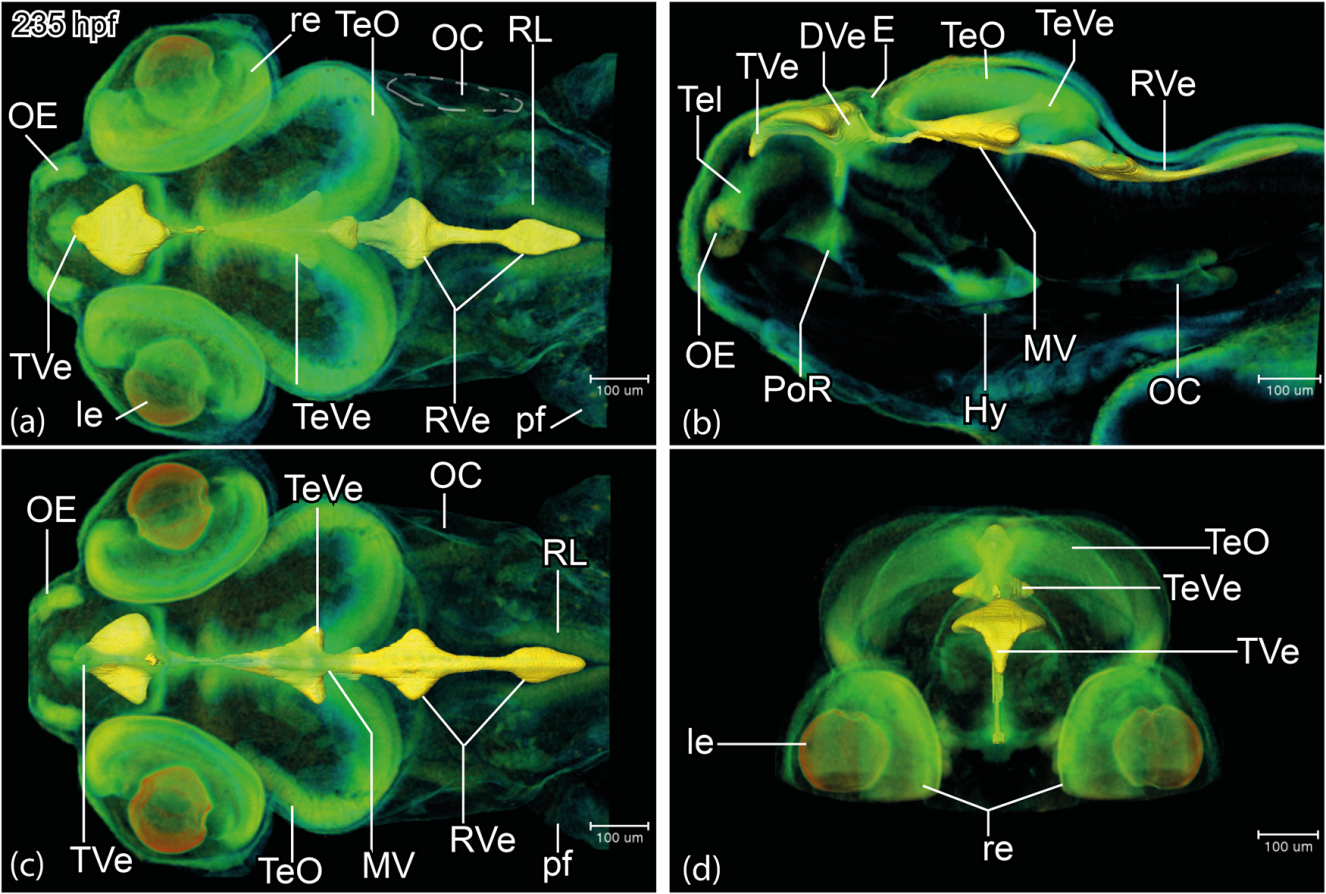
*Rhodeus ocellatus*, brain ventricular system at the stage long‐pec, 235 hours postfertilization (hpf). (a‐d) microCT images, the pseudocolour volume rendering of the head region is overlayed with a surface view of the manually segmented brain ventricles. (a‐d) Dorsal, lateral, ventral, and rostral views respectively. For annotations, see list of abbreviations. Scale bars = 100 μm

The RVe can be divided into two portions, a rostral rhomboid opening with very thin roof plate tenting over, and a caudal elongated ventricle between the rhombic lips (RL; Figure 6a). The rostral view of the TVe gradually deepens from a triangle into a T‐shape (Figure 6d). Compared to earlier stages, the TeVe is further compressed and separated from the MV (Figure 6b). The LR extends more basalward (compare Figure 2c and d), and the IL is enlarged considerably. The PR extends alarward (Figure 2d).

### 3.2 Developmental atlas of the rosy bitterling brain

**FIGURE 7:**
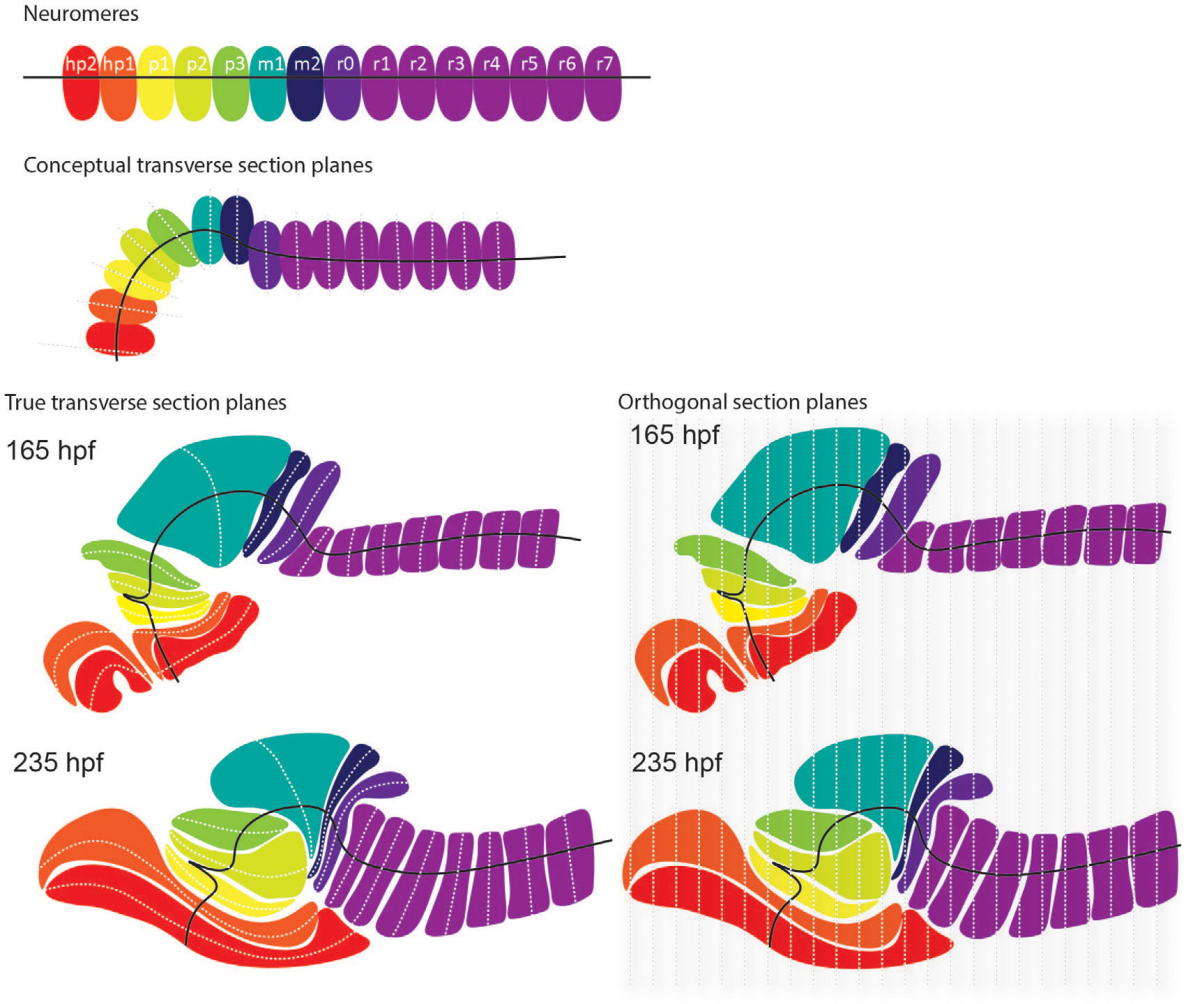
*Rhodeus ocellatus*, comparison of conceptual true transverse section planes and orthogonal section planes. The neuromeres are color‐coded based on the prosomeric model. The black solid line represents the neuraxis, the transverse section planes in the model are marked with white dash lines

**FIGURE 8:**
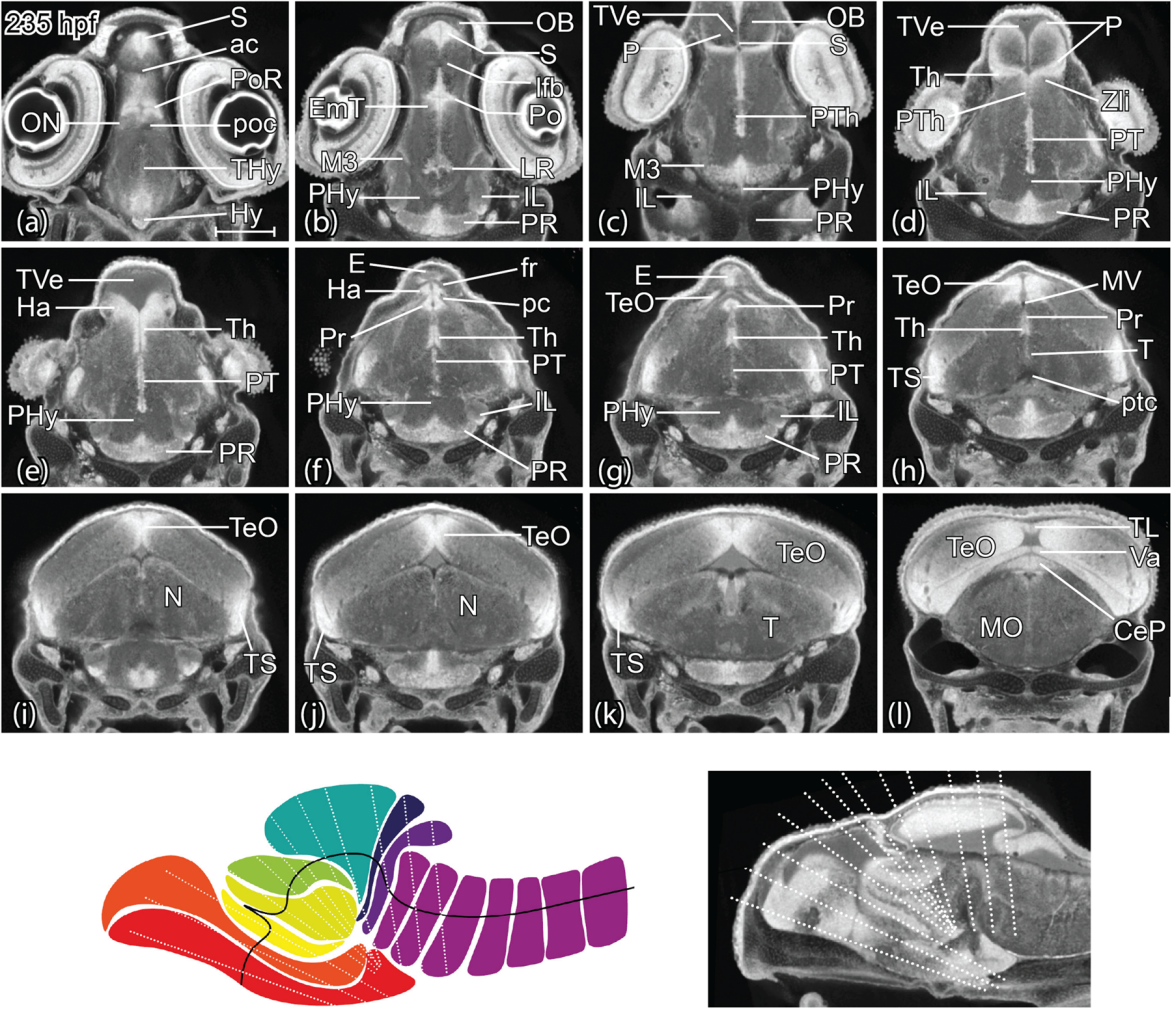
*Rhodeus ocellatus*, brain anatomy, stage long‐pec, 235 hours postfertilization (hpf). (a‐l) microCT images, virtual sections, section plane perpendicular to the neuraxis, dorsal toward the top, direction of section plane indicated in prosomeric model and midsagittal section. For annotations, see list of abbreviations

In addition to the standard topographical cross‐sections of the developing rosy bitterling brain, we also generated “topological cross‐sections” along the anteroposterior axis. In this way, we have provided an additional “prosomeric” perspective, which was not possible with the classical histological techniques. To visualize topological relationships of the neuromeres, we first made conceptual profiles of “topological” transverse sections based on the assumption that each neuromere had at least one representative virtual slice perpendicular to the neuraxis (Figure 7). However, our results show that no section satisfactorily visualizes these arc‐shaped neuromeres, unless they were displayed in nonlinear cutting planes (Figure 7). Due to the flexed neuraxis, “topological” transverse sections do intersect, which inevitably leads to redundant information, best‐exemplified in the case of the hypothalamic region (Figure 8). Virtual sectioning in this way requires constantly reorientating the virtual slices, which is why selecting the cutting plane became quite subjective. In addition, the brain undergoes torsion and rotates along its primary axis during development, which causes an unstable section scheme between the stages. Therefore, we choose the serial orthogonal “virtual transverse section” to display the atlas.

**FIGURE 9:**
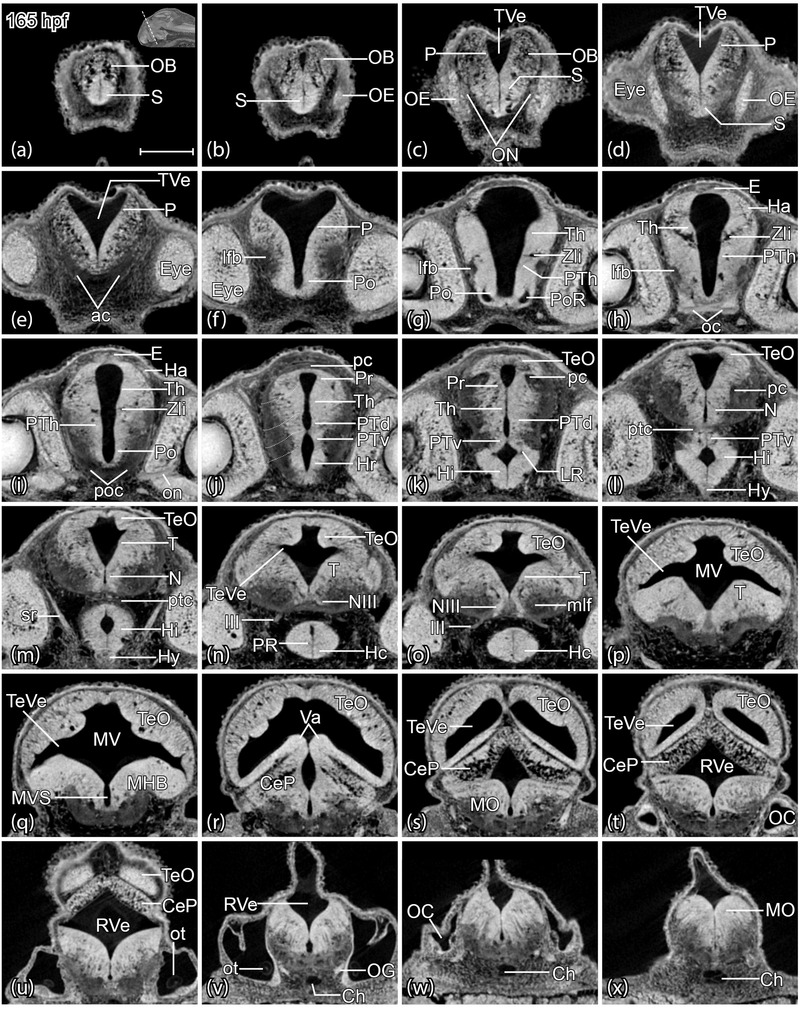
*Rhodeus ocellatus*, brain cross‐sectional anatomy, stage 1‐ovl/pec‐bud, 165 hours postfertilization (hpf). (a‐x) microCT images, virtual sections, transverse plane, dorsal toward the top, sections from rostral to caudal, direction of section plane indicated in inset in (a). For annotations, see list of abbreviations. Scale bars = 100 μm

We noticed that there were cell clusters in periventricular locations that appeared brighter than the adjacent gray matter (e.g., Figure 9j). The distribution of these cell clusters was highly consistent with the distribution of proliferation zones detected during neurogenesis in the zebrafish (Mueller & Wullimann, 2005, 2016). This fact has allowed us in this study to map proliferation zones of the developing bitterling brain and delineate brain territories. Therefore, the delineation of anatomical structures is based on three types of observation: (1) topological relationship to proliferation zones; (2) relative location to annotated brain ventricles (see previous section) and other landmarks, such as commissures and fiber tracts; (3) gray scale values in virtual slices. For example, the zona limitans intrathalamica (Zli) can be demarcated by its dark appearance from the surrounding bright white thalamic tissues (Figure 9g,h).

#### 3.2.1 Secondary prosencephalon

**FIGURE 10:**
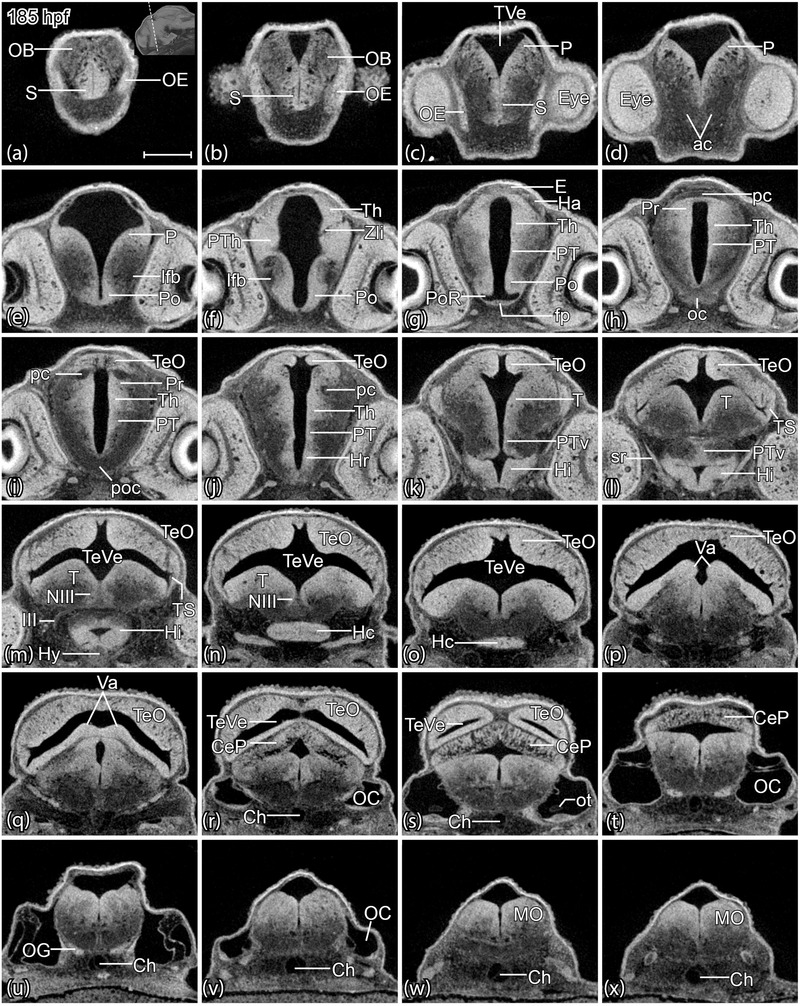
*Rhodeus ocellatus*, brain cross‐sectional anatomy, stage pec‐bud, 185 hours postfertilization (hpf). (a‐x) microCT images, virtual sections, transverse plane, dorsal toward the top, sections from rostral to caudal, direction of section plane indicated in inset in (a). For annotations, see list of abbreviations. Scale bars = 100 μm

**FIGURE 11:**
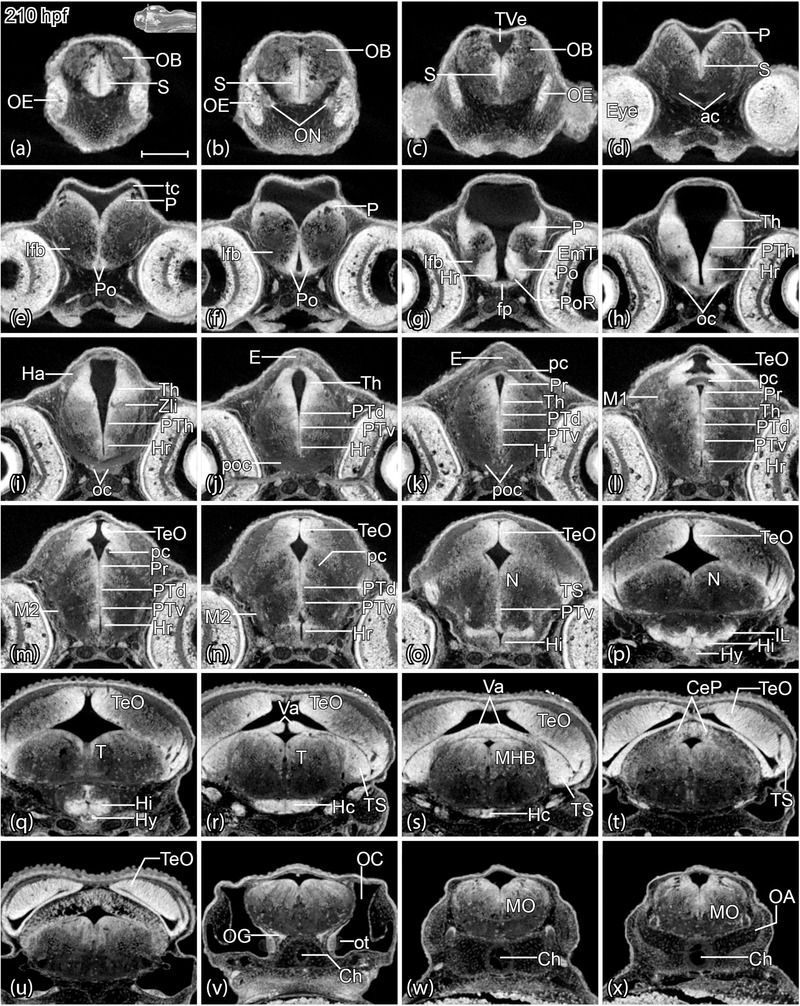
*Rhodeus ocellatus*, brain cross‐sectional anatomy, stage high‐pec, 210 hours postfertilization (hpf). (a‐x) microCT images, virtual sections, transverse plane, dorsal toward the top, sections go from rostral to caudal, direction of section plane indicated in inset in (a). For annotations, see list of abbreviations. Scale bars = 100 μm

**FIGURE 12:**
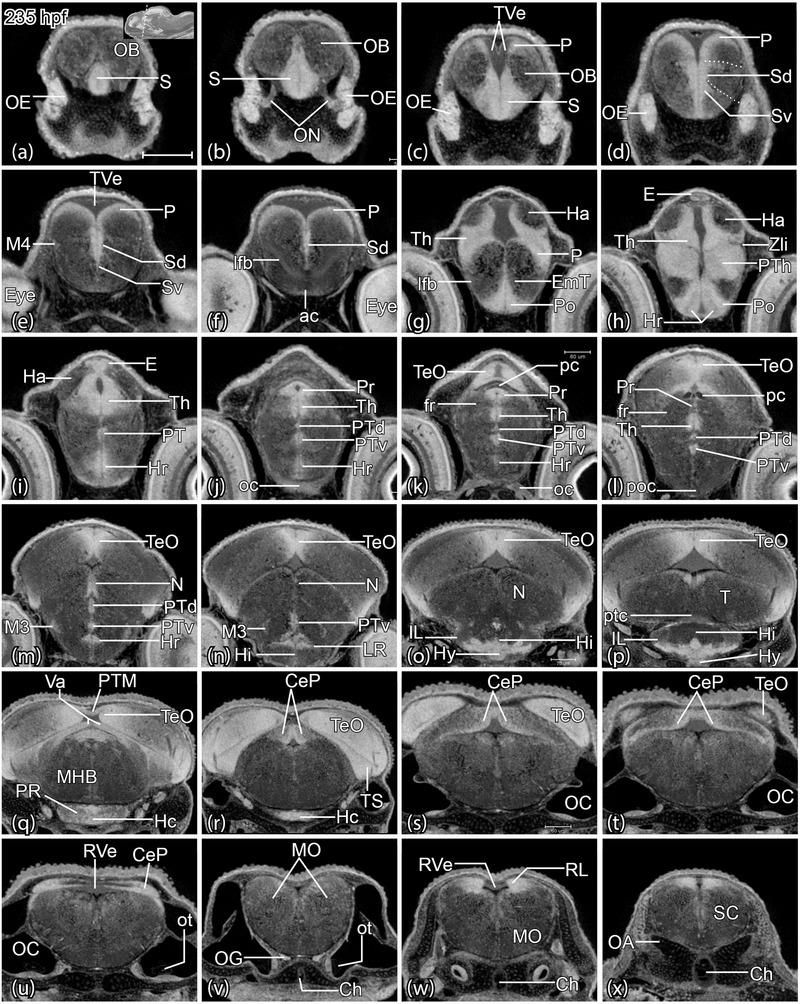
*Rhodeus ocellatus*, brain cross‐sectional anatomy, stage long‐pec, 235 hours postfertilization (hpf). (a‐x) microCT images, virtual sections, transverse plane, dorsal toward the top, sections go from rostral to caudal, direction of section plane indicated in inset in (a). For annotations, see list of abbreviations. Scale bars = 100 μm

Rostral to the telencephalic region, the olfactory epithelium (OE) is very bright, clearly visible from the 1‐ovl/pec fin stage (165 hpf, Figure 9a). At the long‐pec stage (235 hpf), the olfactory epithelium develops into a bow‐shaped structure surrounding the lumen of the olfactory pits (Figure 12a). The OE is connected to the olfactory bulb (OB) through the easily recognizable olfactory nerve (ON; Figures 9, 11, and 12b). The OB is characterized by its glomerular structure (Figures 9, 10, 11a and 12a; Dynes & Ngai, 1998).

The TVe is located at the dorsum of the pallium. The tela choroidea is in the roof of the ventricle. The periventricular proliferation zones of the subpallium (S) and pallium (P) in our samples appeared distinctively bright in virtual transverse sections. At the long‐pec stage (235 hpf), the bright subpallial cell clusters were separated by distinct, dark boundaries (dashed line in Figure 12d), corresponding to the dorsal and ventral subdivisions of the subpallium (Sd and Sv). The zones of pallial proliferation consist of a few cell rows, and are visible as a pair of arches flanking the dorsal subpallium (Figures 9, 10, 11d, and 12c). The telencephalic migrated area (M4) was recognizable as nonventricular proliferation cell clusters at the margin of the lateral subpallium (Figure 12e).

Immediately caudal to the subpallium, the ac and the lateral forebrain bundle (lfb) appeared as a dark fiber bundle crossing the rostral end of the forebrain (Figures 9e,f, 10d,e, 11d,e, and 12f). In this study, we define the ac and the poc as the boundaries of the preoptic region (Po) in this study. Recently, this region has also been considered the alar hypothalamus (aHyp) or the ORR (Affaticati et al., 2015; Schredelseker & Driever, 2020; Slack, 2005). The proliferation zone of the Po was easy to identify based on is triangular shape, which surrounded the optic recess. The Po proliferation zone was broad on its ventral side, thinning out dorsally (Figures 9, 10, 11e, and 12g). The optic chiasma (oc), which has a bright, thick appearance, decussates in the midline (Figures 9, 10, 11h, and 12j); it marks the topologically anterior end of the neural axis.

In the basal hypothalamic region, the hypophysis topologically located acroterminal (anterior) to the hypothalamus (Figure 2). It projects from the ventral midline of the brain (Figure 2). As in the zebrafish developmental brain atlas, we divided the basal hypothalamus into the following regions: (i) the intermediate hypothalamus (Hi) near the hypophysis and including the IL; (ii) the rostral hypothalamus (Hr) near the rostral end; and (iii) the caudal hypothalamus (Hc) near the caudal end. The hypothalamus encloses the hypothalamic ventricles including the LR in the Hi and PR in the Hc. This division is consistent with those classically used for describing the zebrafish hypothalamus (Wullimann et al., 1996; Manoli & Driever, 2014; Biran et al., 2015; Mueller & Wullimann, 2016; Muthu et al., 2016).

However, it should be noted that recent molecular studies in zebrafish improved the comparative interpretation of the teleostean hypothalamus and its evolutionary relationships with the mammalian hypothalamus (Baeuml et al., 2019; Herget et al., 2014). Likewise, gene expression in the PR tuberal region of embryonic zebrafish revealed homology with two domains of the mammalian hypothalamus, the TuV (tuberal region, ventral part) and TuI (tuberal region, intermedia part). Therefore, Schredelseker and Driever (2020) proposed to refer to this region as posterior recess region (PRR) to show its teleost‐specific phylogeny. Due to the fact that our analysis in the rosy bitterling is based on purely anatomical microCT data, however, we were not able to relate our findings with the recent ones. For this purpose, future studies are required that analyze appropriate gene expression patterns in bitterlings.

#### 3.2.2 Diencephalon

The prosomeric model divides the diencephalon, from caudal to rostral, into alar and basal plate derivatives. In this model, the pretectum (aP1), the thalamus proper (aP2), and the prethalamus (aP3) form the alar plate portions of the diencephalon (Lauter et al., 2013). In contrast, the proliferation zones of the nucleus of the medial longitudinal fasciculus (N; bP1), and the dorsal (bP2) and ventral (bP3) posterior tegmentum (PT; Mueller & Wullimann, 2005, 2016) form the corresponding basal plate derivatives.

Along the roof plate of the diencephalon, the most prominent structure is the epiphysis (E), a swelling in the dorsal midline of the brain (Figures 9, 10, 11j, and 12i). The habenular nuclei (Ha) are located one each side of it (Figures 9, 10, 11i, and 12i) and show discrete cell clusters from the pec‐bud stage (185 hpf) onward (Figure 10g). Notice that the fasciculus retroflexus (fr) appeared in our microCT photographs in the form of distinctive, dark fiber‐bundles in the gray matter (Figure 12k,i). It originates from the Ha and connects to the interpeduncular nucleus across isthmus (r0) and rhombomere 1 (Akle et al., 2012). In the prosomeric model, the fasciculus retroflexus is used to delimit pretectum (P1) and thalamus (P2; Akle et al., 2012; Lauter et al., 2013; Puelles, 2019). We use the caudal end of the pc as the caudal boundary of the P1, which divides the diencephalic area and the mesencephalic area (Figures 9, 10, 11l, and 12k).

In the thalamic region, we identified the zona limitans intrathalamica (Zli) as a dark band (Figures 9, 10, 11i, and 12h) that marks out the boundary between prethalamus (P3) and thalamus (P2). Therefore, we annotated the separate periventricular proliferation zones of prethalamus (PTh) and thalamus (Th) in the transverse virtual section based on their topological relationships (anterior vs. posterior) and Zli landmark (e.g., Figure 12j).

The thalamic eminence (EmT) is a relatively complex region in the diencephalon and most often viewed in the prosomeric model as the anterior portion of the PTh (hence often termed “prethalamic eminence,” PThE). However, while the EmT (or PThE) generates glutamatergic derivatives, the PTh proper forms predominantly GABAergic territories. In addition, some recent studies in zebrafish and tetrapods indicate that the Emt/PThE contributes to telencephalic territories such as the medial extended amygdala and newly identified nucleus of the lateral olfactory tract (Alonso et al., 2020; Porter &and Mueller, 2020; Vicario et al., 2017). Due to the lack of molecular expression patterns, we stayed conservative in our analyses and placed the EmT topologically anterior to the PTh and posterior to the preoptic region, similar to the one that has been described for larval zebrafish (Wullimann & Mueller, 2004). It abuts the lfb (Mueller, 2012), which is identifiable in our CT scans (Figures 9, 10, 11e, and 12f). Molecular markers such as Tbr‐1 are needed to validate our annotations (Wullimann, 2009; Wullimann & Mueller, 2004).

Note that the PT has distinct PTd and PTv proliferation zones (e.g., Figure 12k). The neural axis is flexed here (i.e., at the cephalic flexure), and so the dorsal‐ventral topology, in the virtual transverse sections, actually corresponds to the anterior‐posterior axis of the neural tube.

#### 3.2.3 Mesencephalon and rhombencephalon

The boundary between the diencephalon and mesencephalon defined dorsally by the posterior end of the pc and ventrally by the anterior margin of the oculomotor nerve root (Moreno et al., 2016). In the tegmentum, we were able to identify the oculomotor nerve (III), which typically projects ventrolaterally from the oculomotor nucleus (NIII) and exits at the ventral surface of the brain (e.g., Figure 9n,o). The proliferation cluster of the NIII, and the basal plate of the mesencephalon, are thereby demarcated.

In the prosomeric model, the mesencephalon contains two mesomeres, m1 and m2, from anterior to posterior. The tectal gray, optic tectum (mammalian superior colliculus), and torus semicircularis (mammalian inferior colliculus) constitute the alar plate of m1. The NIII represents the basal plate of m1.

We noticed two pairs of tectal membrane thickenings that invaginate into the TeVe toward the tegmentum at the 1‐ovl/pec‐bud stage on 165 hpf (Figure 9q,r). During development, the boundaries between these thickenings gradually disappears as they grow together (Figures 10o and 11o). The tectal proliferation zones are distinct. At the 1‐ovl/pec‐bud stage (165 hpf) and the pec‐bud stage (185 hpf), the tectal region appears as a large, bright field with the microCT (Figures 9o and 10l). Beginning with the high‐pec stage (210 hpf), the rostral tectal proliferation becomes restricted to one mediodorsal cluster and two bilateral clusters (Figures 11o and 12n). These lateral and medial proliferation zones merge in the midline at caudal levels and form a continuous cap of tectal proliferation (e.g., Figure 11t).

**FIGURE 13:**
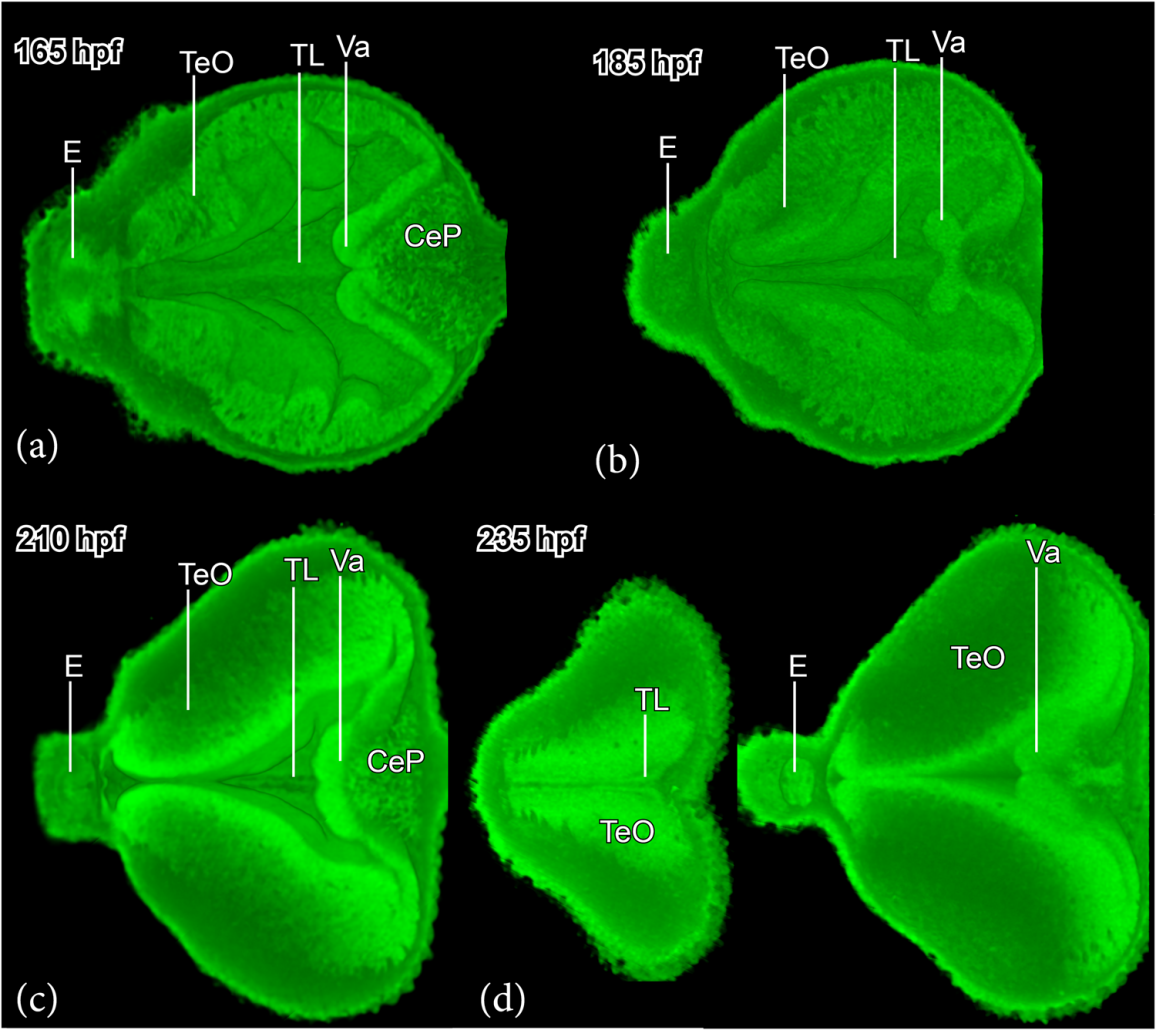
*Rhodeus ocellatus*, development of the torus longitudinalis. Virtual dissection, head to the left. (a) Stage 1‐ovl/pec‐bud, 165 hours postfertilization (hpf). (b) Stage pec‐bud, 185 hpf. (c) Stage high‐pec, 210 hpf. (d) Stage long‐pec, 235 hpf. For annotations, see list of abbreviations

The torus longitudinalis (TL) is a specialized brain region exclusive to ray‐finned fish (Folgueira et al., 2020; Wullimann, 1994). We identified the TL from rostral to caudal along the medial margins of the optic tectum. Virtual horizontal sections through the optic tectum, from the level of epiphysis and the rostral cerebellar thickening, revealed that the TL can be seen at the top of the MV (Figure 13).

**FIGURE 14:**
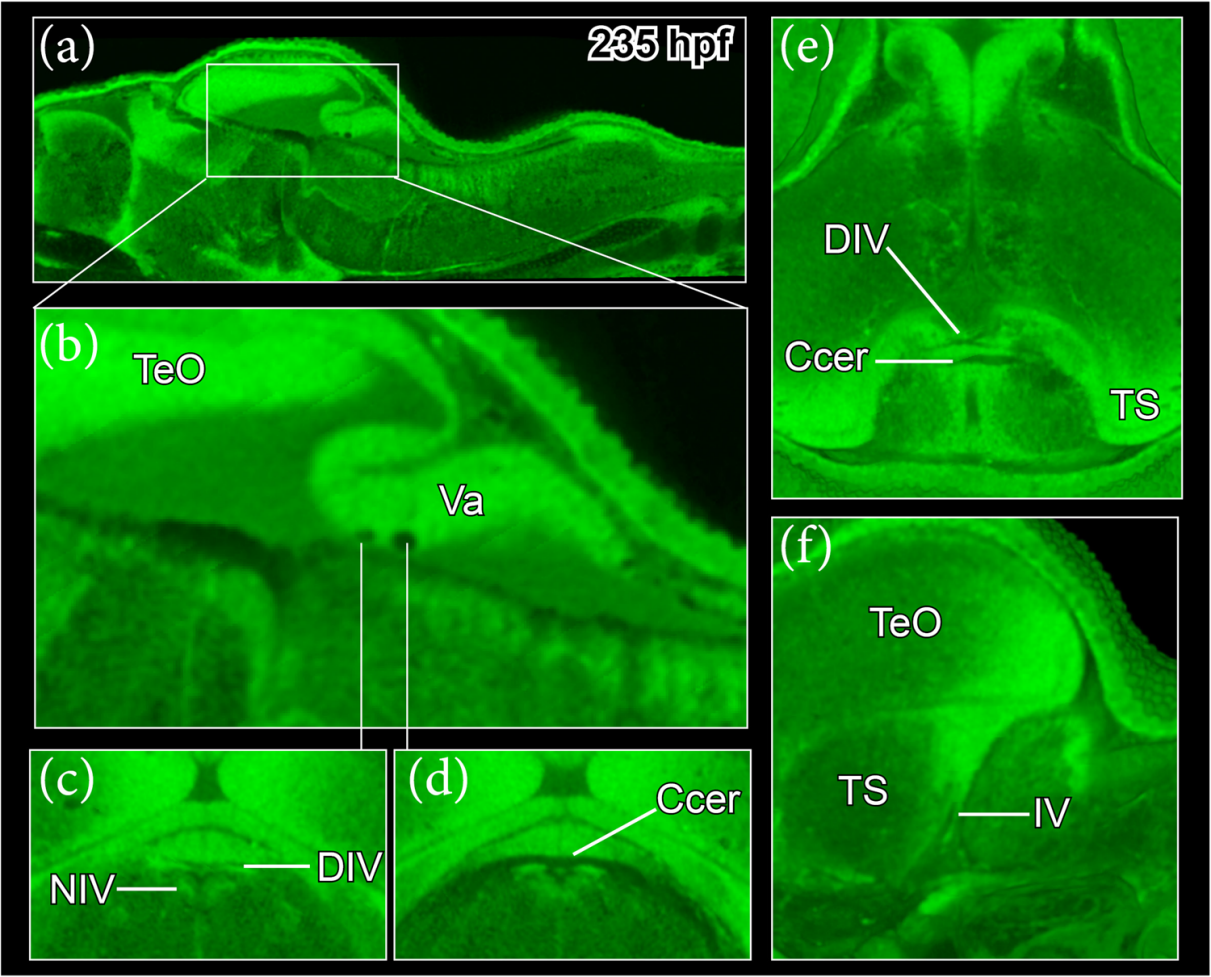
*Rhodeus ocellatus*, trochlear nerve, stage long‐pec, 235 hours postfertilization (hpf). (a‐f) microCT images, virtual sections. (a and b) Midsagittal plane, head to the left, dorsal toward the top. (c and d) transverse plane, dorsal toward the top. (e) Horizontal plane, head toward the top. (f) Parasagittal plane, head to the left, dorsal toward the top. For annotations, see list of abbreviations

The r0, or isthmus, is at the midbrain‐hindbrain boundary (MHB) region. The MHB is composed of the posterior tectal membrane and the rostral cerebellar thickenings (the valvula cerebelli; Wullimann & Knipp, 2000); it appeared bright throughout the developmental period (Figures 9, 10, 11r, and 12q). The cerebellar plate appeared bright only at its basal and medial aspects (e.g., Figure 12t,u). The trochlear nucleus (NIV) topologically belongs to r0. It is easier to identify the trochlear decussation (DIV) and the commissure cerebelli (Ccer) in the midsagittal section (Figure 14a‐d) in the valvula cerebelli (Va). Then, follow the caudolateral projection of the trochlear axon in the horizontal section (Figure 14e) until it exits the brain as the trochlear nerve (IV, Figure 14f) between torus semicircularis and rhombencephalon. The axon tract of the trochlear nucleus delineates the boundary between r0 and r1.

**FIGURE 15:**
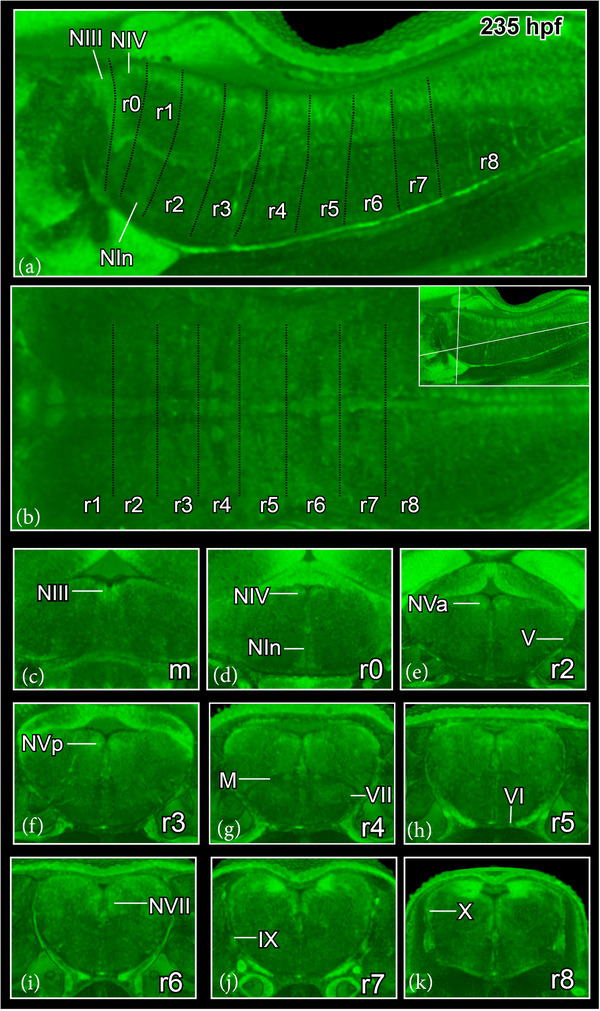
*Rhodeus ocellatus*, hindbrain segmentation, stage long‐pec, 235 hours postfertilization (hpf). (a) microCT images, virtual sections, midsagittal plane, dorsal toward the top, head to the left. The black dash line indicates the rhombomeric boundaries. (b) Horizontal plane, head to the left, direction of section plane indicated in inset at the upper right corner. (c‐k) Transverse plane, dorsal toward the top, sections go from the rostral to caudal, direction of section plane indicated in inset in (b). For annotations, see list of abbreviations

The fasciculus retroflexus (fr) of teleosts innervates the interpeduncular nucleus (NIn). Thus, we used the white matter tract of the fr to identify the NIn in the bitterling embryo (Figure 15a). According to Lorente‐Cánovas et al. (2012), the NIn is at the basal plate across isthmus (r0) and r1. The r1 is devoid of cranial motor neurons (Nieuwenhuys & Puelles, 2016); therefore, we used the posterior margin of the NIn as a landmark for the boundary between r1 and r2.

In the more caudal rhombencephalic region, the roof plate is a thin layer of tela choroidea, which is visible in microCT scans because it remains intact during the procedure (e.g., Figure 9x). The dorsal medullary proliferation zone is broad and expands ventrally up to the high‐pec stage on 210 hpf (Figures 9, 10, and 11v), but at the long‐pec stage (235 hpf) it becomes more restricted, forming the rhombic lip proliferation zone (Figure 12w).

**FIGURE 16:**
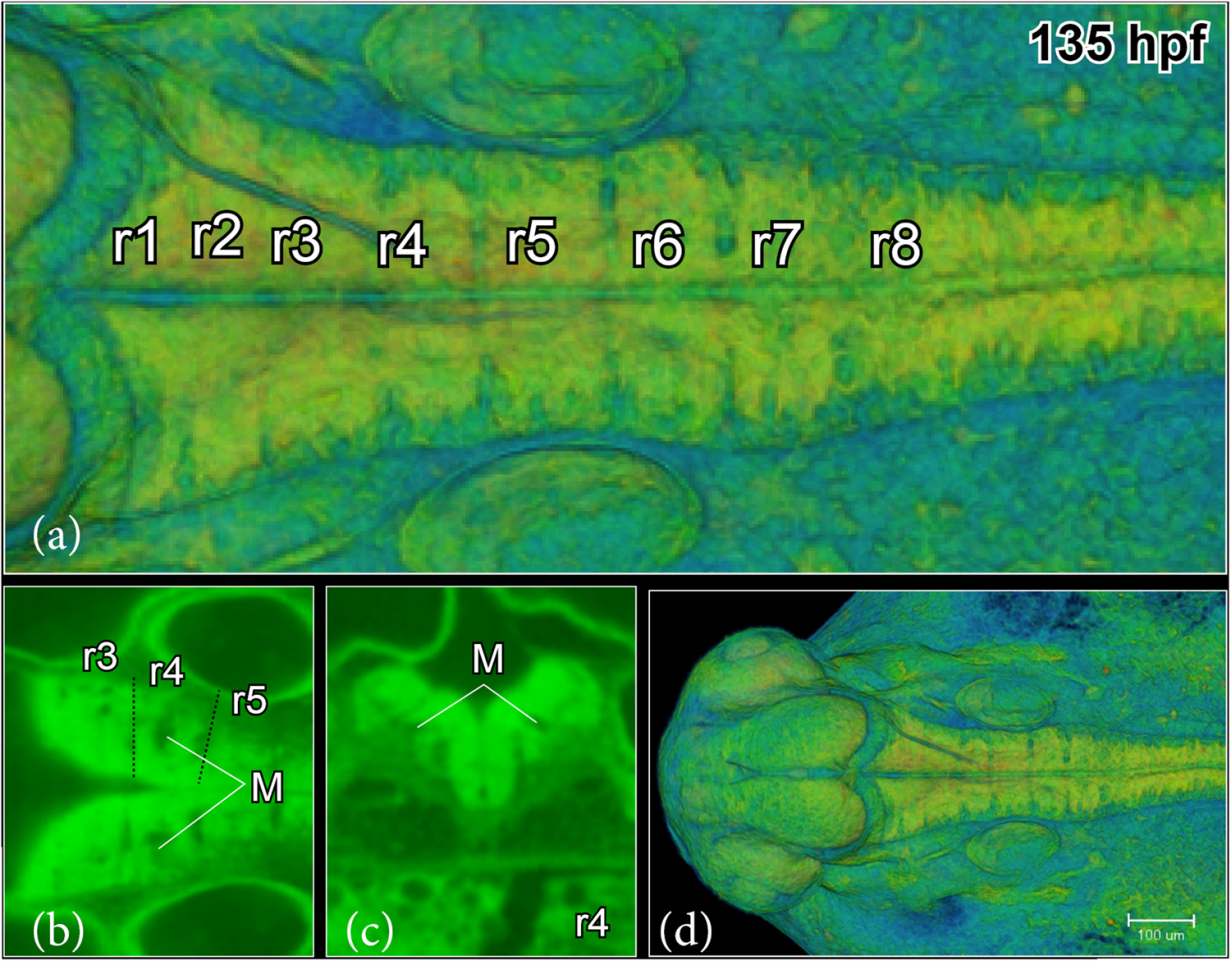
*Rhodeus ocellatus*, hindbrain segmentation, 135 hours postfertilization (hpf). (a and d) microCT images, volume rendering, show boundaries between rhombomeres. (b) Virtual section, horizontal plane, head to the left, show the location of the Mauthner cell at the centre of r4. (c) Virtual section, transverse plane, dorsal toward the top. For annotations, see list of abbreviations

The boundaries between rhombomeres from r2 to r8 are visible in early bitterling embryos at 135 hpf (Figure 16), but soon become less visible from the 1‐ovl/pec fin stage at 165 hpf onwards. However, teleost fish retain a segmented pattern of reticulospinal neurons through embryonic stage to adulthood (Gilland et al., 2014). For example, the large bilateral Mauthner neurons (M) are the marker of r4 (Eaton & Farley, 1973; Moens & Prince, 2002). By slicing the embryo of 135 hpf at the transverse level of r4, it is determined that the Mauthner cell resides near the central of rhombomere 4 rather than at the segmental boundaries (Figure 16). The rhombomeric segments of the older embryo (235 hpf, Figure 15a‐k) were thereby identified by assuming that each reticulospinal neuronal cluster is located in the center of each rhombomere.

The nerve roots of the cranial nerves are also reliable landmarks of rhombomeres. By tracing the projection of cranial nerves, we identified the trigeminal nerve root (V) in r2 (Figure 15e), the facial (VII) and the accompanying vestibulo‐cochlear nerves in r4 (Figure 15g), the abducens root (VI) in r5 (Figure 15h), the glossopharyngeal root (IX) in r7 (Figure 15j), and the vagus root (X) in r8 (Figure 15k). The r2 was also labeled by somata of the anterior trigeminal motor neuron (NVa, Figure 15e) and the r3 by the posterior trigeminal motor neuron (NVp, Figure 13f). The facial motor neuron (NVII) is distinct in r6 (Figure 15i).

## 4 DISCUSSION

### 4.1 Brain imaging and 3D visualization of neuroanatomy

To gain insight into the complex morphogenesis of the bitterling brain, we analyzed the formation of the brain ventricular system in 3D from the stage 1‐ovl (150 hpf) to long‐pec (235 hpf) of bitterling development. We combined annotations of brain functional subdivisions and morphological landmarks, based on microCT scanning results and on the literature for the zebrafish embryo. We found that 3D visualization with microCT scanning protocols was extremely useful for the study of the rosy bitterling and provides an updating of imaging modalities for morphological and anatomical analyses in this nonmodel organism. A systematic application of microCT may offer an essential foundation for future comparative studies of the teleost brain.

Our study provides 3D reconstruction of brain ventricles in the bitterling, showing similar organization to the zebrafish larval ventricular system as visualized by dye‐injection into the hindbrain ventricle (Lowery & Sive, 2005). We have also defined boundaries of brain ventricle subdivisions based on published anatomical landmarks (Turner et al., 2012). Furthermore, the flexure of the neuraxis was easily visualized continuously from the rostral tip of the brain to the spinal cord. Understanding this cephalic flexure is crucial for the correct topological interpretation of the brain, especially with regard to the highly complex secondary prosencephalon (Hauptmann & Gerster, 2000; Puelles, 2019). Studies have shown that the mechanisms of brain ventricle development are highly conversed across vertebrates (Lowery & Sive, 2009). The CSF in the brain ventricular system could contribute to specialization of the early brain because the production and flow of CSF performs an important role in homeostasis of the CNS (Fame et al., 2016).

In contrast to other vertebrates, actinopterygian fish‐like teleosts lack a pair of lateral ventricles in the telencephalon (Wullimann & Rink, 2002). Instead, they show a T‐shaped midline TVe that is the result of a morphogenetic process called “eversion” (Mueller & Wullimann, 2009). Our 3D models showed that in the rosy bitterling, the AIS develops much earlier than the eversion of the TVe, and expansion of the dorsal ventricular surface of the AIS is synchronous with the eversion process. Our results are consistent with the TVe morphogenesis described in zebrafish (Folgueira et al., 2012). During early embryogenesis, we were able to visualize the generation of the deep ventricular sulcus (AIS) followed by an anterolateral eversion of the telencephalic neuroepithelium.

We found distinct periventricular cell clusters in the gray matter with higher grayscale values than the surrounding tissue. Their distribution pattern was highly consistent with the distribution of proliferation zones described during neurogenesis of zebrafish (Mueller & Wullimann, 2003, 2016; Mueller et al., 2006; Wullimann, 2009; Wullimann & Knipp, 2000; Wullimann & Mueller, 2004). It is possible, for example, that newly postmitotic neurons appear brighter than most of the postmitotic cell masses in more peripheral positions, remote from the periventricular proliferation zones. The result of immunohistochemistry for the neurotransmitter GABA (γ‐aminobutyric acid) in zebrafish (Mueller & Wullimann, 2016; Mueller et al., 2006; Panganiban & Rubenstein, 2002) and PCNA (proliferation cell nuclear antigen) for proliferation patterns (Wullimann &and Knipp, 2000; Wullimann & Mueller, 2004; Wullimann & Puelles, 1999) is consistent with our interpretation of the proliferation zones.

This study paves the way to more detailed analyses of the bitterling brain. We hope that it will also prove valuable in studies using the growing number of fish models (and even non‐fish models). Application of microCT 3D imaging provides a heuristic model of the brain, an extremely complex anatomical region. Importantly, our approach is validated by the fact that the profile of CT values displayed here in the bitterling brain is consistent with genoarchitecture identified in previous neurodevelopmental studies. For example, our annotation of the zona limitans intrathalamica (ZLI) is extremely close to the highly conserved expression pattern of the gene sonic hedgehog (*shh*; Vieira et al., 2005; Mueller et al., 2006; Scholpp et al., 2006). In addition, microCT allows time‐efficient imaging of intact brains while providing a resolution (micron level) sufficient for displaying critical landmarks, groups of neurons (such as proliferation zones versus postmitotic cell masses), and white matter tracts. These histological characteristics are critical for detecting landmarks and visualizing structural features as a means of describing developmental neuroanatomy. However, the resolution of microCT is inferior either to light microscopy of histological brain sections or to light sheet microscopy‐based imaging of fluorescence‐stained intact brains. It is also lower resolution than Synchrotron imaging, whose resolution reaches the submicron level and therefore permits quantitative histological phenotyping (Ding et al., 2019). Neurons and neuropils in the CNS can be selectively stained with the salts of metallic elements, including gold, silver, platinum, and mercury chloride (Keklikoglou et al., 2019; Mizutani & Suzuki, 2012), which can compensate for the inability to use antibodies in combination with microCT. In summary, MicroCT imaging produces lower resolution than some imaging modalities, but has the special advantage that complex 3D models can be produced without the need for time‐consuming reconstruction from histological material. The specimens imaged with microCT can be subsequently run through paraffin histology if needed.

### 4.2 Comparison between developmental stages of bitterling and zebrafish

The rosy bitterling and the well‐studied zebrafish are closely related (Cypriniformes: Cyprinidae; Mayden et al., 2009). Their phylogenetic relationship allows for a relatively straightforward interspecies comparison of their brain development. Likewise, the process of brain ventricle inflation, flexion of the neuroaxis, and establishment of prosomeric units happens in the bitterling at comparable stages to the zebrafish. They do, however, differ in absolute timing. Specifically, the 165 hpf bitterling brain and 30 hpf zebrafish brain are in the same process of brain ventricle inflation and have a similar degree of flexion of the neuroaxis. We noticed that stratification of the proliferation zones of the diencephalic region (Pr, Th, PTd, PTv) is consistent between the 185 hpf bitterling brain and 36 hpf zebrafish brain.

**FIGURE 17:**
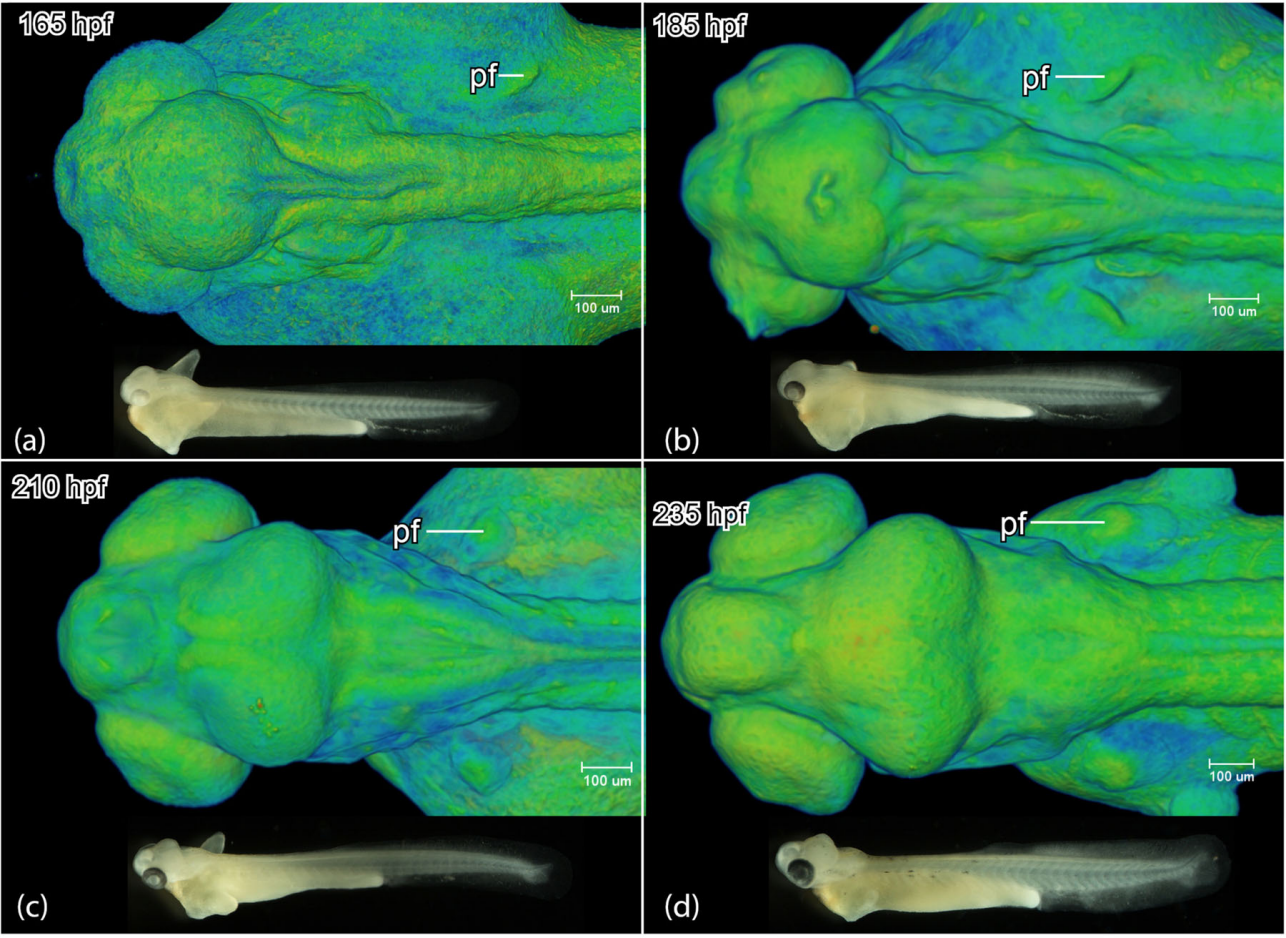
*Rhodeus ocellatus*, development of the brain region and the pectoral fin bud. Dorsal view of the head region microCT images, pseudocolored volume‐renderings, rostral to the left. Lateral view of the embryo, photomicrographs, rostral to the left. (a) 1‐ovl/pec‐bud, 165 hours postfertilization (hpf); (b) pec‐bud, 185 hpf; (c) high‐pec, 210 hpf; (d) long‐pec 235 hpf. For annotations, see list of abbreviations

However, distinct developmental heterochronies have been detected between bitterling and zebrafish brains. The bitterling shows precocious development of the inferior lobe and basalward extension of the LR in the hypothalamic region (Figure 2): the inferior lobe is remarkable so early at the long‐pec stage (235 hpf in bitterling, Figure 17; 48 hpf in zebrafish). The long‐pec stage is defined by the degree of development of the pectoral fin bud, and is defined when the bud height grows to twice the width of the bud base (Kimmel et al., 1995; Yi et al., 2021). In zebrafish, the inferior lobe has not developed at the long‐pec stage; it is first identifiable at 5 days postfertilization (Bloch et al., 2019). In teleost fish, the inferior lobe is known as a multisensory integration center that is involved in gustatory (Rink & Wullimann, 1998; Wullimann, 2020), visual (Butler et al., 1991), and octavolateralis systems (Yang et al., 2007). Further studies can use the detailed descriptions of the bitterling brain developmental atlas provided here to uncover the underlying regulatory mechanisms that control such heterochronic development.

## AUTHOR CONTRIBUTIONS

Wenjing Yi and Michael K. Richardson conceived the study. Wenjing Yi performed all neuroembryology studies, including microCT analysis. Martin Rücklin helped with microCT studies. All authors collaborated with Wenjing Yi on interpreting the microCT data. Wenjing Yi prepared the manuscript and figures. Michael K. Richardson and Thomas Mueller helped in editing the manuscript.

## CONFLICT OF INTEREST

The authors declare that they have no competing interests.

### PEER REVIEW

The peer review history for this article is available at https://publons.com/publon/10.1002/cne.25324.

## Data Availability

The data of this study are available from the corresponding author upon reasonable request.
